# Assessing adaptive requirements and breeding potential of spelt under Mediterranean environment

**DOI:** 10.1038/s41598-021-86276-1

**Published:** 2021-03-30

**Authors:** Arie Y. Curzon, Chandrasekhar Kottakota, Kamal Nashef, Shahal Abbo, David J. Bonfil, Ram Reifen, Shimrit Bar-El, On Rabinovich, Asaf Avneri, Roi Ben-David

**Affiliations:** 1 Department of Vegetable and Field Crops, Institute of Plant Sciences, Agricultural Research Organization (ARO)-Volcani Center, 7528809 Rishon LeZion, Israel; 2 The Levi Eshkol School of Agriculture, The Hebrew University of Jerusalem, 7610001 Rehovot, Israel; 3 Department of Vegetable and Field Crops, Institute of Plant Sciences, Agricultural Research Organization (ARO)-Gilat Research Center, 8531100 Rishon LeTsiyon, Israel; 4 The School of Nutritional Sciences, The Robert H. Smith Faculty of Agriculture, Food and Environment, The Hebrew University of Jerusalem, 7610001 Rehovot, Israel; 5 Northern R&D, P.O. Box 831, 11016 Kiryat Shmona, Israel

**Keywords:** Plant breeding, Plant sciences, Agricultural genetics

## Abstract

The rising demand for spelt wheat (*Triticum aestivum* ssp. spelta) as a high-value grain crop has raised interest in its introduction into non-traditional spelt growing areas. This study aimed to assess adaptive constrains of spelt under short Mediterranean season. At first screening of a wide spelt collection for phenology and allelic distribution at the photoperiod (*PPD*) and vernalization (*VRN*) loci was done. In addition an in-depth phenotypic evaluation of a selected panel (n = 20) was performed, including agronomically important traits and concentration of grain mineral (GMC) and grain protein (GPC) content. Results from both wide screening and in-depth in panel (group of 18 spelt lines and two bread wheat lines) evaluation shows that the major adaptive constraint for spelt under Mediterranean conditions is late heading, caused by day length sensitivity, as evident from phenology and allelic profile (*PPD* and *VRN*). All lines carrying the photoperiod-sensitive allele (*PPD-D1b*) were late flowering (> 120DH). Based on the panel field evaluations those consequently suffer from low grain yield and poor agronomic performances. As for minerals, GMC for all but Zn, significantly correlated with GPC. In general, GMC negatively correlated with yield which complicated the assessment of GMC *per-se* and challenge the claim for higher mineral content in spelt grains. The exceptions were, Fe and Zn, which did not correlate with yield. Spelt lines showing high Fe and Zn concentration in a high-yield background illustrate their potential for spelt wheat breeding. Improving spelt adaptation to Mediterranean environments could be mediated by introducing the insensitive-*PPD-D1a* allele to spelt wheat background. Following this breeding path spelt could better compete with bread wheat under short season with limited and fluctuating rain fall.

## Introduction

Spelt wheat (*Triticum aestivum* ssp. spelta) is a hulled cultivar group with a lax 'speltoid' spike, that belongs to the species of bread wheat (*T. aestivum*), traditionally considered to carry a distinct gene pool^1^. In the past, spelt was widely cultivated in northern Europe, but, over recent centuries, it has been replaced by higher-yielding (free-threshing) bread wheat germplasm. However, recently, spelt is enjoying a growing demand from consumers, bakers, and farmers^2^ and presented as 'healthy alternative' to bread wheat^3^.

It is presumed that spelt has a higher protein concentration^1^ and higher mineral concentration than bread wheat, particularly, higher Fe, Zn, Cu, Mg and P levels^4^. Yet, a recent study showed that spelt germplasm is genetically diverse for grain protein and minerals such as Zn and Fe^5,6^ and is therefore considered a highly promising source for genetic diversity of these traits.

Spelt and bread wheat differ in spike morphology and threshing characteristics. Bread wheat has a dense compact spike, in spelt the spike is lax. Bread wheat is free-threshing, while spelt lines are hulled and the chaff is only released in an additional mechanical process^7^. These attributes and other spike characteristics are thought to be mainly controlled by a single pleiotropic gene (*Q*, chromosome 5A)^8,9^. A recessive *q* alleles acquires the spelt phenotype, while bread wheats semi-dominant *Q* allele causes a free-threshing character. In addition, Spelt and Bread wheat differ in more attributes such as the grain shape. Spelt grains are longer and less round in comparison to bread wheat grains^7^. This might be a result of a pleiotropic effect of spike morphology and structure, as shown by Millet^10^. A recent study suggested that the *Q* gene might also influence the naked grain characteristics^11^.

Phenology is the major adaptive component of rain-fed field crops in any growing environment^12^. In wheat, flowering is mainly controlled by the response to vernalization and photoperiod, both of which provide possibilities for environmental adaptation^13^. The response to vernalization is attributed to polymorphism in *VRN1* genes (5A, 5B, 5D), dividing wheats into winter and spring types, with and without vernalization requirements, respectively. On the other hand, the response to photoperiod is controlled by the *PPD1* genes (2A, 2B, 2D), dividing wheats into photoperiod-sensitive and -insensitive types, with and without long-day requirements, respectively^14–17^. Wheat is the main dryland field crop in Israel and its yield and quality highly vary with precipitation and temperature^18–20^. Rain-fed spring wheat is sown at the onset of the mild winter (late November) and grows throughout the season, reaching heading before terminal drought in the spring (March). In contrast, spelt wheat, which has mainly been cultivated in northern high latitudes areas in Europe^2^, with long photoperiod and cold temperatures, requires genetic modification in phenology-related traits in order to improve its adaptation to Mediterranean environments.

With a focus on the adaptation of spelt wheat to Mediterranean environments, the following study aimed to (1) assess spelt adaptation flexibility to Mediterranean conditions, (2) compare spelt wheat *vs.* bread wheat grains for mineral and protein content and (3) identify spelt breeding targets in order to optimize its agronomic performance under Mediterranean environment.

## Results

### Evaluation of a wide spelt collection phenology and plant height

Based on two field seasons (2015–16, 2016–17), the spelt germplasm was late-heading as compared to local (Israel) spring wheat cultivar checks Ruta and Shefa (Figure S1b,d). Out of 182 lines tested, only a few were early-heading (DH < 120 days). The few identified early-heading spelt lines were also found to be free-threshing, unlike the rest of the spelt lines, but similar to the bread wheat checks. Similarly, spelt plant height (Figure S1a,c), which was widely distributed, was generally much higher than the spring wheat checks (PH < 1 m). Genotyping with PPD and VRN1 markers clearly shows that all late heading genotypes carry *PPD-D1b* sensitive allele. Allelic profiles at the *VRN1* and *PPD1* genes of the germplasm lines showed that most genotyped spelt lines were spring types (Supplementary Table S2) but were photoperiod-sensitive (*PPD-D1b*), explaining the late heading of these lines in comparison to Israeli wheat checks (Supplementary Table S2). Only a very limited number of spelt lines (Supplementary Table S2). PI 520066, TAS03, 109, carried a day length-insensitive (*PPD-D1a*) allele among those were the earliest spelt lines  grown in this study.

### Sowing time effect on onset of heading

**Figure 1:**
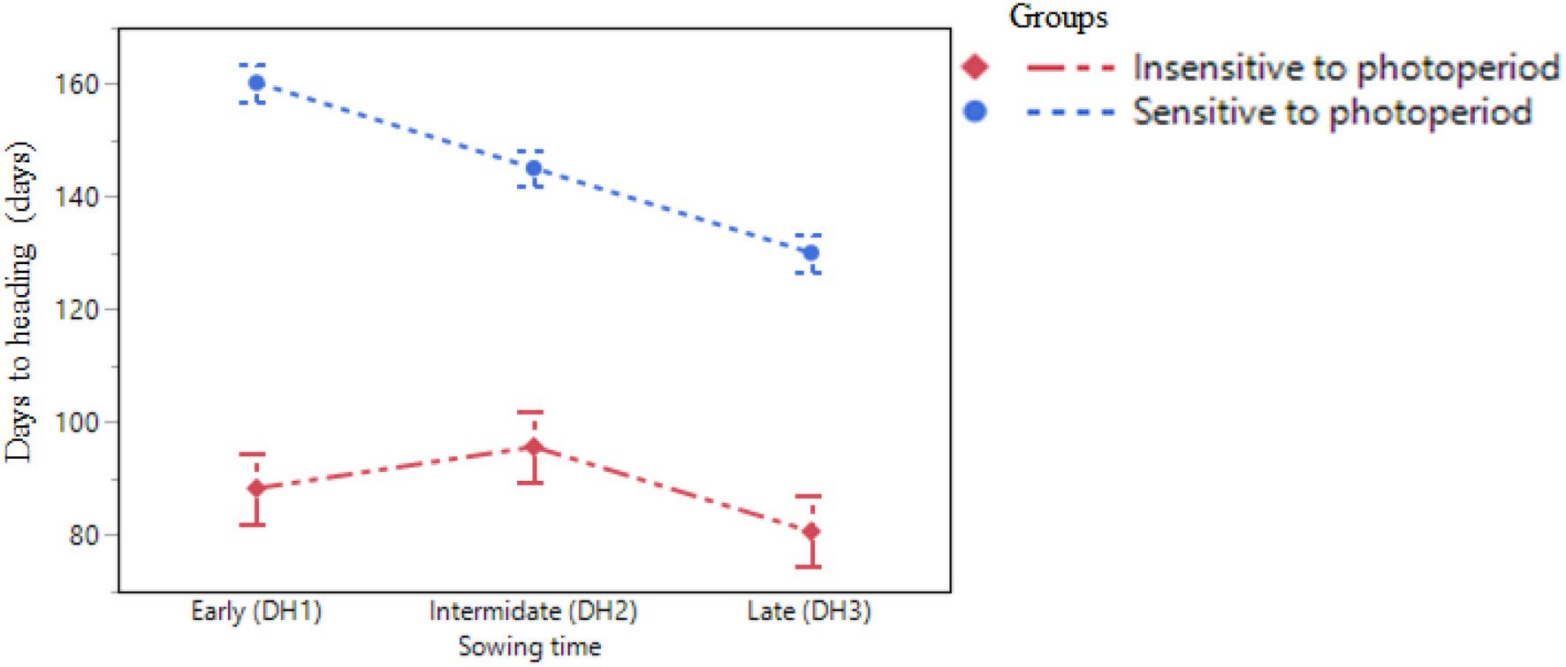
Days to heading of photoperiod-sensitive and insensitive genotypes sown on different dates. Mean (± SE) days to heading of two groups photoperiod-sensitive (PS, blue line, carrying the *PPD-D1b* allele, Supplementary Table S2) and photoperiod-insensitive (PIS, red line, carrying the *PPD-D1a* allele, Table S2) for early—8/11/15, intermediate—23/11/15 and late -12/12/15 sowing dates.

Although there was an approximate two-week sowing date interval (SDI) between sowing dates and approximately 30 days between the earliest and latest sowing date (Supplementary Table S3), in most cases, the heading date interval (HDI) for each line was shorter than one week. The majority of spelt genotypes headed around the same date (Fig. 1), regardless of sowing date (early, mid or late). These lines were also characterized as photoperiod sensitive (carrying the *PPD-D1b* allele, Supplementary Table S2). Out of the fifteen lines in the panel (# marked in table S1), four exceptions were found to this pattern, *i.e*., the two spring wheat checks (cv. Ruta and Shefa) and two spelt lines TAS03 and 1152. Ruta, Shefa and TAS03 were characterized as photoperiod-insensitive (carrying the *PPD-D1a* allele, Supplementary Table S2) the HDI between the first and second sowing cycles with larger HDI than the gap between the second and third cycles. HDI between the first and last sowing cycle was longer than one month (Supplementary Table S3 and Fig. 1). For line 1152, the effect of the sowing date on onset of heading was more moderate, with six days between HD of each sowing cycle (only 12 days between the first and third). These four lines were significantly earlier to head than the other lines grown in 2015–16. In general, heading dates among lines in the core panel had similar trends in both growing seasons (2015–16 and 2016–17), as phenology grade (from early to late heading) across the lines was similar (Table 1, Supplementary Table S3).

**Table 1:** Mean data from the 20 lines grown in replicates experiment 2016–17 Bet-Dagan, Israel.

Line	Gene bank ID	DH	Yield (g)/0.25m2	DM (g)/0.25m2	HI	TKW(g)	Hull (%)	SC	GS	PH (cm)	SW (cm)
–	CGN06533	145	55	479	0.23	35.5	9.4	1.60	2.7	140	2.8
–	CGN08306	139	88	655	0.29	55.4	0.2	2.10	2.2	149	4.5
TAS03	–	103	99	373	0.44	61.7	0.4	1.40	2.3	111	4.6
–	CGN08295	152	54	579	0.22	39	61.3	1.60	2.7	140	3.1
TAS06	–	148	38	368	0.23	41	50.7	1.60	2.8	134	3
Tomarense	PI 608792	141	95	852	0.25	53.1	0.7	1.40	2.2	157	4.5
69Z5.142	PI 355661	148	53	450	0.26	40.6	37	1.50	2.6	142	3.4
109	PI 225295	147	87	756	0.25	43.1	2.5	1.40	2.1	161	3.8
1772–14,038	PI 191617	148	43	418	0.23	37.7	51.2	1.60	2.7	134	3.4
1755	PI 378480	150	88	820	0.24	48.2	26.5	1.50	2.5	175	4.4
1152	PI 367203	120	124	678	0.35	49.3	4	1.70	2.3	140	3.3
Rojo	PI 191100	152	64	598	0.23	46.7	36.9	1.60	2.3	165	4.3
White Spring	PI 168682	150	62	629	0.23	39.2	59.3	1.70	2.6	136	3.3
2670	PI 190962	151	45	402	0.24	36.1	62.4	1.60	2.8	127	3
Canadian	–	151	59	545	0.24	34	43.6	1.60	2.3	117	3.7
Oren	–	166	27	357	0.18	31.7	42.1	1.60	2.5	108	3.4
–	CGN08309	149	30	336	0.19	33.8	70.6	1.60	2.6	148	3
Saharense	–	109	96	491	0.37	47.5	5.3	1.90	2.0	136	4.4
Shefa	–	110	85	275	0.48	43.5	0.5	2.00	1.9	84	4.5
Ruta	–	113	103	355	0.47	37.1	0.6	2.1	1.9	98	5
S.E		0.4	9.4	63.06	0.01	1.7	2.5	0.3	0.01	4.5	0.2
R square		1	0.75	0.71	0.91	0.88	0.97	0.44	0.52	0.89	0.8
Prob > F		< 0.001	< 0.001	< 0.001	< 0.001	< 0.001	< 0.001	0.01	< 0.001	< 0.001	< 0.001
F Ratio		1666	8.44	7.21	34	23	103	2.16	322	24	12
MS		1272	3010	114,795	0.02	254	2656	0.68	22	1960	1.85
SS		24,169	57,185	2,181,107	0.62	4830	50,469	13	416	37,236	35
DF		19	19	19	19	19	19	19	19	19	19

Days to heading (DH), Yield (g), Dry matter (DM), harvest index (HI), thousand kernel weight (TKW), weight percentage of hulled seeds (Hull (%), spike compactness (SC), grain shape (GS), plant height (PH), stem width (SW).—: unknown.

### Genotypic evaluation of the panel

DNA markers screening of the core spelt panel comprehensively characterized in the field during 2016–17, showed that the three lines with the early phenology (TAS03, Shefa and Ruta) were spring types. This was determined based on screening of the *VRNA1*, *VRNB1* and *VRND1* DNA markers. Each of these early heading genotypes carried spring-type alleles in at least two of these three loci and an insensitive allele at *PPD-D1a* (Supplementary Table S2), accounting for their earliness and insensitivity to day length. No photoperiod-insensitive allele was detected in 1152, explaining its relative lateness (heading at 24/02/16 when day length is 11 h + 19 min). Similarly, the line Saharense, which headed early (Table 1) and possessed spring-type alleles (Supplementary Table S2) was not found to carry any day-length insensitive allele. Both 1152 and Saharense seems to possess a certain degree of insensitivity to day length, but the actual genetic locus causing this, remains unidentified in these lines. The only line (out of the fifteen lines-core panel) with an allelic profile that combined complete winter type (vernalization alleles) and photoperiod sensitivity (Supplementary Table S2) was Oren spelt, which was also the last to flower among the tested lines (2016–17, Table 1). Line 109 was found to be a winter type, with a day-length insensitive *PPD-D1a* allele (Supplementary Table S2), and to head late, illustrating that the spring type allele is also required in order to secure early phenology under Israeli growth condition, as winter temperatures cannot satisfy the vernalization requirements.

**Figure 2:**
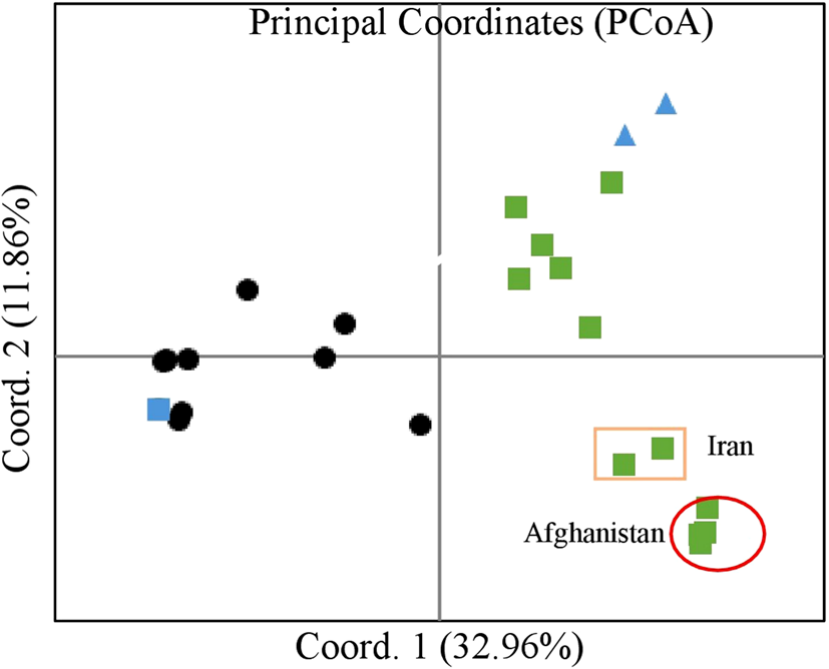
Principal Coordinate analysis of 26 lines Genotyped by 83 KASP markers representing the AABB genome. Panel of 24 spelt lines and two bread wheat cultivars (see supplementary S1 labelled as star mark). Modern bread wheat (blue triangle) Free-threshing spelt (green rectangle) Hulled spelt (filled circle). GenAlEx 6.503^47^ was used to calculate binary genetic distances between genotypes. Principal coordinate analysis (PCoA) was used to illustrate genetic distances.

The PCoA analysis (Fig. 2) of 26 lines showed a separation between hulled and free-threshing lines based on 83 KASP markers representing the AABB genome. This is illustrated along the horizontal axis (Coord. 1) that represents most of the genetic variance (32.96%). The two bread wheat checks clustered together, but other free-threshing lines had a large genetic range, mainly along the vertical axis (Coord. 2) which represented less than 12% of the variance (Fig. 2). Interestingly, four free-threshing lines which were collected together in Afghanistan [PI 367200, PI 367201, PI 367202 and PI 367203(1152)], clustered together at the right end of the chart along with two lines from Iran (Fig. 2), showing their common genetic relatedness.

### Core-panel in-depth phenotypic evaluation

**Figure 3:**
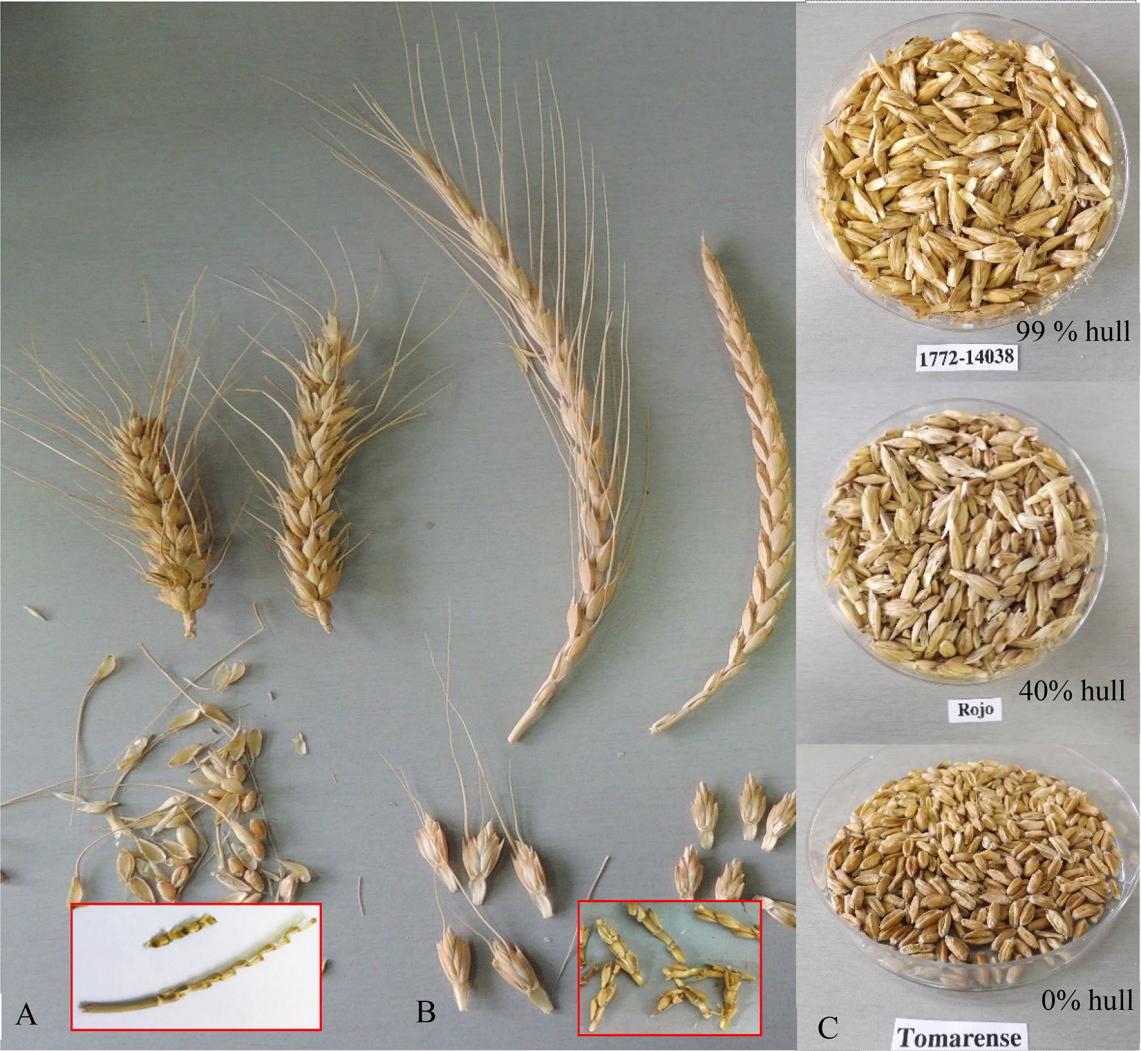
Spike morphology and threshing characteristics of (**A**) Bread wheat, complete spike (upper), threshed spike (middle) and rachis (highlighted red colour square) (**B**) Spelt complete spike (upper), threshed spike (middle) and Rachis (highlighted red colour square) and (**C**) Spelt hullness 1772–14308 (PI 191617) around 99% hullness (upper), Rojo (PI 191100) around 40% hullness (middle), Tomarense (PI 608792) free threshing (lower).

Most of the spelt lines in the panel had a ‘lax spike’, with low spike compactness, in comparison to the bread wheat checks, supporting their initial gene bank definition as spelt (Table 1). Although spelt lines are characterized as being hulled, several lines described as spelt by the gene bank, were free-threshing. However only two of these lines, Saharense and CGN08306, showed a combined phenotype of a dense spike as well (Table 1). The two lines were also characterized by high stature (PH > 1.35 m) and could be referred to as bread wheat landraces. Thus, although originally thought to be controlled mainly by the same gene, *Q*, spike morphology (expressed by the calculated SR ratio) and hullness did not significantly correlate (Table 2). This explains the screening results, which identified five free-threshing lines with a speltoid spike. Still, in most cases, there was a difference between the threshing outcomes of these free-threshing speltoid lines and free-threshing bread wheat lines, as illustrated by the tougher spelt glumes, which remained partly connected to the rachis after mechanical threshing (Fig. 3).

**Table 2:** Pearson correlation matrix of phenotypic and yield components measured in a block design experiment 2016–17.

	DH	Yield (g)	DM (g)	HI	TKW (g)	Hull (%)	SC	GS	Plant height (cm)	SW (cm)
DH	#									
Yield	− 0.74***a	#								
DM	0.22	0.44	#							
HI	− 0.94***	0.70***	− 0.29	#						
TKW	− 0.54*	0.71***	0.41	0.43	#					
Hull (%)	0.69***	− 0.83***	− 0.24	− 0.68***	− 0.63***	#				
SC	− 0.49*	0.23	− 0.3	0.62***	0.00	− 0.33	#			
GS	0.69***	− 0.75***	− 0.11	− 0.74***	− 0.41	0.77***	− 0.56**	#		
Plant height	0.40	0.05	0.74***	− 0.56***	0.32	0.08	− 0.62***	0.27	#	
SW	− 0.60**	0.69***	0.17	0.67***	0.60***	− 0.69***	0.37	− 0.83***	− 0.11	#

Days to heading (DH), Yield (g), Dry matter (DM), harvest index (HI), Thousand kernel weight (TKW), weight percentage of hulled seeds (Hull %), Spike compactness (SC), Grain shape (GS), Plant height (PH), Stem width (SW). a Confidence interval is marked * < 0.05 ** < 0.01 *** < 0.001.

**Figure 4:**
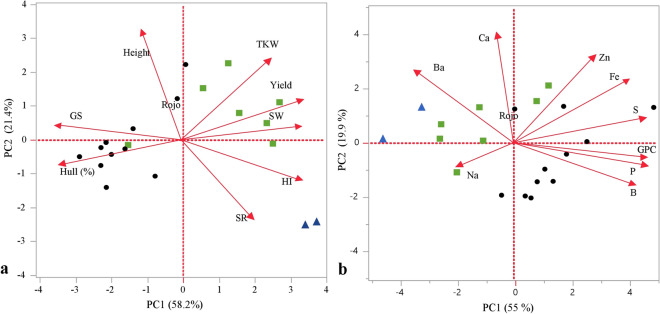
Principal components analysis of agronomic traits and mineral concentration of 20 lines grown under field conditions. Biplot vectors are trait factor loadings for PC1 and PC2. (**a**) Agronomic traits of Stem width (SW), Spike compactness (SC), Grain shape (GS), Thousand-kernel weight (TKW), Hull (%), grain yield (GY), Harvest index (HI) (**b**) Mineral concentration in ppm of Barium (Ba), Boron (B), Calcium (Ca), Iron (Fe), Phosphorus (P), Sodium (Na), Sulphur (S), Zinc (Zn) and Grain protein concentration (GPC). (blue triangle) modern bread wheat (green rectangle) free-threshing spelt (filled cirecle) hulled spelt.

As illustrated in Fig. 4a, the two checks bread wheat lines were different from the other spelt lines, mainly in SR, height and HI (highly influenced by height), with both exhibiting a dense spike and short stature in comparison to the rest of the lines. In general, hulled lines (with more than 10% hull after threshing) were late to head and had lower GY, TKW and HI [(*P*) t < 0.001 for all traits] (see also Table 2 for correlations among traits and Fig. 4a). Hulled lines were also characterized by longer grains (higher GS values) and thinner stems expressed in lower SW [(*P*) t < 0.001 for all traits]. This is illustrated in Fig. 4a and described in detail in Tables 1 and 2. In terms of GY free threshing lines and modern checks were superior comparing to hulled lines which was also expressed by a pronounced negative correlation with GY (− 0.83). Late supplementary irrigation applied at the end of March was contributing to grain filling of lines with intermediate phenology but not to the early heading modern checks (which headed three weeks earlier and almost completed grain-fill). Consequently, correlation value between DH and GY was lower than expected (-0.74) under Mediterranean conditions. DH had the strongest correlation with HI (− 0.94) and this is not confounded by PH or DM, which were not significantly correlated with DH or GY.

The five selected spelt lines grown in the field for three successive seasons, showed grain yield ranges of 138–347 g/m^2^, 188–329 g/m^2^ and 15–185 g/m^2^ for the 2016–17, 2017–18 and 2018–19 seasons, respectively (Supplementary Figure S3 present mean yield from the two later seasons). Grain yield fluctuated between seasons, as demonstrated by line 1152, which showed a relative high GY in 2017–18, but the lowest GY of 2018–19 season. This might be explained by severe yellow rust and powdery mildew infection accompanied with significant lodging. White spring cultivars showed yield stability in all years (~ 200 g/m^2^), as expressed in low P values (Supplementary Figure S3). TKW of spelt kernels was only evaluated in 2017–18 and exhibit a range of 61.5–94.8.

### Comparative evaluation of grain mineral and protein concentration

The 20 lines in the germplasm panel (Supplementary Table S1, * marked samples) were tested for GMC and GPC. When comparing the two modern bread cultivars to the tested spelt lines, it was noticeable that, in general, they had a lower concentrations of B, Cu, Fe, K, Mg, Mo, P, S, Si, Sr and Zn, but higher concentrations of Ba and Ca (Fig. 4b). However, when comparing all the lines to the bread wheat ‘Ruta’, these differences were not significant for all tested lines in the panel (Supplementary Table S4). Similarly, significant differences were observed for hulled and free-threshing lines for mineral and grain protein concentrations (Fig. 4b and Supplementary Table S5), due to a generally higher value of grain minerals and protein concentrations in hulled lines (Supplementary Table S5). A clear diagonal separation between the two-modern bread wheat lines, Ruta and Shefa, and between free-threshing (relatively high Ba and Ca) and hulled lines (high GPC, P, S and B) was observed with one outlier, the hulled wheat line Rojo (PI 191100) which plotted within the free-threshing cluster (Fig. 4b).

**Figure 5:**
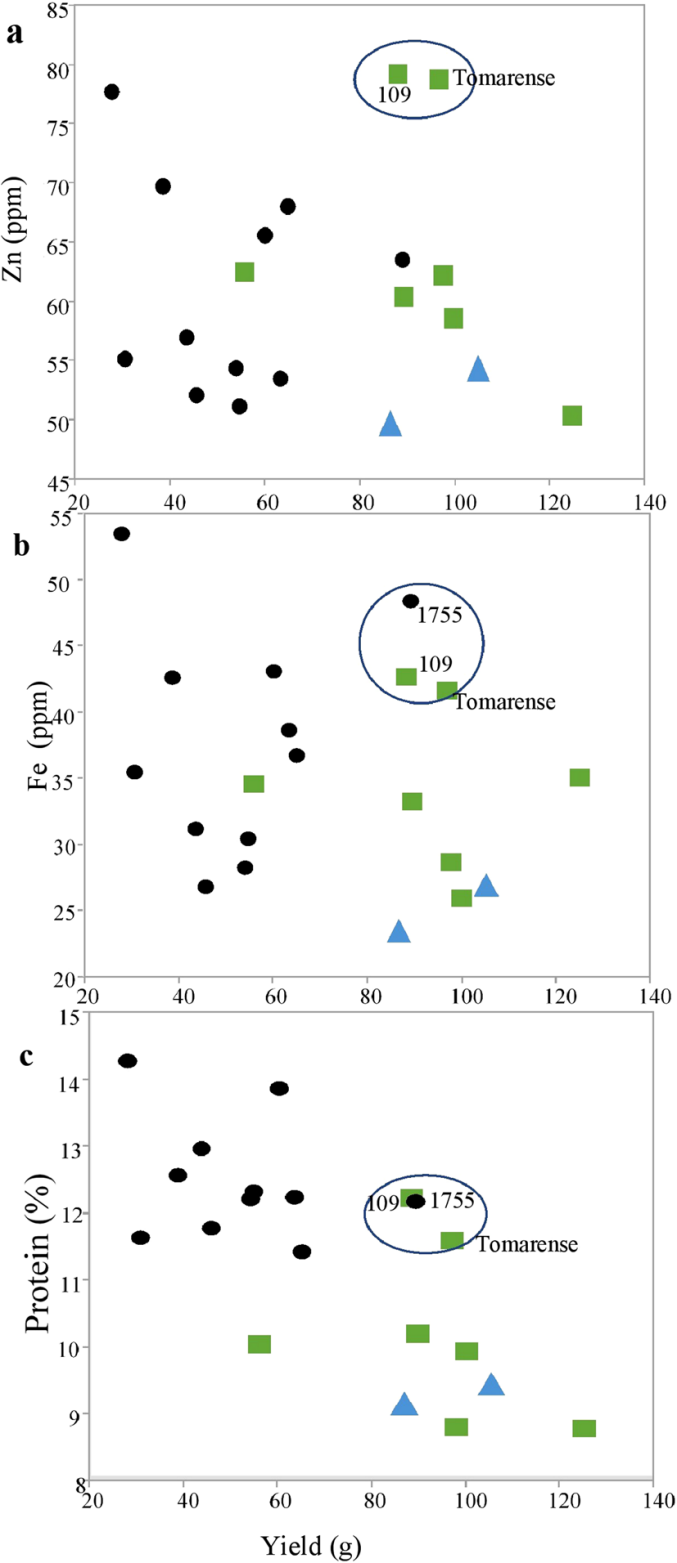
Grain Mineral Concentration and Grain Protein Content compared to Grain Yield. Mean values of Zn, Fe and protein concentration plotted against yield of the 20-line block design (n = 4) experiment (Panel: P4). Mineral concentration (ppm) by inductively-coupled plasma mass spectrometry. Modern bread wheat (blue triangle) Free-threshing spelt (green rectanagle) Hulled spelt (filled circle). The samples marked in blue line circles show high yield and exceptionally high concentrations of Zn and Fe and high GPC values. Tomarense and 109 (Free threshing) showed high values for all three parameters. 1755 (Hulled) did not show an exceptionally high concentration for Zn.

In general, parameters of mineral concentrations significantly positively correlated with GS and negatively correlated with GY (especially the macronutrients N, P, K, and S) exceptions were the Zn, Fe, Si, and Na concentrations, which were not significantly correlated with GY and TKW (Table 3 and Fig. 5). The negative correlation with GY explains why the mineral content of the hulled lines (concentration multiplied by GY) showed a completely opposite trend to mineral concentration (Table S5). With no exception free-threshing lines had significantly higher mineral content in comparison to hulled lines. Fe, like most of the tested minerals, correlated significantly with P, S and GPC. In contrast, Zn, despite its significant correlation with S, showed only a weakly significant correlation with GPC and did not correlate significantly with P. Interestingly Zn, GMC and GPC were not significantly correlated (*p* < 0.068).

**Table 3:** Pearson correlation conflict of mean GMC^a^, GPC (%), Yield (gr), GS, TKW (gm) measured in a block design experiment 2016–17.

	B	Ba	Cu	Fe	K	Mg	Na	P	S	Si	Zn	GPC	Yield	GS
B	#													
Ba	− 0.71***a	#												
Cu	0.84***	− 0.69***	#											
Fe	0.51*	− 0.36	0.52**	#										
K	0.83***	− 0.59***	0.67***	0.57***	#									
Mg	0.82***	− 0.72***	0.81***	0.69***	0.82***	#								
Na	− 0.14	0.11	− 0.18	− 0.47*	− 0.08	− 0.24	#							
P	0.86***	− 0.74***	0.83***	0.68***	0.88***	0.98***	− 0.20	#						
S	0.69***	− 0.45*	0.77***	0.76***	0.66***	0.79***	− 0.37	0.84***	#					
Si	0.58**	− 0.57**	0.65**	0.70***	0.50*	0.72***	− 0.49*	0.67**	0.60**	#				
Zn	0.21	− 0.13	0.24	0.73***	0.23	0.35	− 0.26	0.37	0.64***	0.24	#			
GPC	0.84***	− 0.61***	0.83***	0.64***	0.74***	0.83***	− 0.27	0.90***	0.90***	0.60**	0.42	#		
Yield	− 0.80***	0.65***	− 0.68***	− 0.24	− 0.68***	− 0.63***	− 0.02	− 0.69***	− 0.54*	− 0.33	− 0.08	− 0.71***	#	
GS	0.59**	− 0.63***	0.62***	0.23	0.72***	0.72***	0.02	0.73***	0.48*	0.46*	− 0.08	0.58***	− 0.75***	#
TKW	− 0.69***	0.51*	− 0.44	− 0.19	− 0.57**	− 0.43	− 0.14	− 0.47*	− 0.23	− 0.23	0.07	− 0.50*	0.71***	− 0.41

^a^GMC: Grain mineral concentration of elements (ppm): Barium (Ba), Boron (B), Copper (Cu), Iron (Fe), Magnesium (Mg), Phosphorus (P), Potassium (K), Silicon (Si), Sodium (Na), Sulphur (S), Zinc (Zn), Grain protein concentration (GPC), Yield (g), and Grain size (GS).

^b^Confidence interval is marked * < 0.05 ** < 0.01 *** < 0.001.

Grain protein concentration was tested in a wider set of lines, including samples of bread wheat lines and spelt lines, amounting to a total of 166 free-threshing samples and 90 hulled samples. As can be seen in Figure S2, GPC showed a wide distribution range in hulled and free-threshing samples, illustrating that spelt and bread wheat do not differ distinctly for these two traits (*p* < 0.65).

## Discussion

### Adaptation of spelt germplasm to the Mediterranean environment

Based on the phenology of the wide collection of spelt lines and the core panel sown on three different dates (2015–16) and monitored in a field experiment (2016–17), it can be concluded that the main limitation for growing spelt in a Mediterranean environment is their inherent late heading (after mid-March). While bread wheat cultivars in Israel headed 84–99 days from emergence, spelt lines reached heading no earlier than 105 days, with the exception of TAS03 and 1152.

Three lines (Ruta, Shefa, and TAS03) did not show a phenology compaction when sown on three different dates (Supplementary Table S1 and Fig. 1). This suggests that they are all, to some extent, photoperiod-insensitive. The three started to head around the beginning of February, when day length is about 10 h + 30 min. The late heading of the other spelt lines suggests that they are photoperiod-sensitive. This conclusion is supported by the above-mentioned heading date 'compaction’ and is underscored by the fact that the earliest heading date (Tomarense, Table 1) of the spelt lines was on 01/04/2017. On this date, day length is already 12 h + 30 min. These findings demonstrate that regardless of the timing of sowing, heading of spelt lines occurred around the same period, suggesting that this response is mediated mainly by day length sensitivity. A broad screening of late flowering spelt genotypes for *VRN1* and *PPD-D1* confirmed that spelts are almost exclusively late-flowering, lacking a photoperiod insensitive allele at *PPD-D1a*. Only one additional line (PI 520,060, in addition to 109 and TAS03 which were already described earlier) was identified in the broad screening as an exceptionally early flowering genotype. Similarly, to the other two this line was found to be insensitive at *PPD-D1a*. The insensitive *PPD-D1a* allele was introduced to wheat cultivars during the “green revolution”^15^, an intensive and wide breeding manipulation that likely skipped over other spelt lines that were grown on marginal lands or abandoned with the adaption of high-yielding bread wheat varieties^2^.

The presented data regarding the allelic profile of the *VRN1* loci might be incomplete, as more complexity was found for *VRN1* alleles^21^, and additional spring alleles for *VRN1* genes were identified (such as:^22,23^). Still, it seems that our dataset suffices to understand the major constrains for spelt phenological adaptation to Mediterranean environment. Although temperature is also highly influential on wheat phenology, we did not evaluate its effect on plant growth and phenology in the current work; the extent of its influence remains open for further evaluation. Accordingly, *earliness *per se alleles were not characterized here and might also affect the final timing of flowering of a given line. Phenology seems to be the main limitation for the adaptation of spelt to Mediterranean environment. Additionally, enhancing grain yield by introduction of *Rht* dwarfing alleles might also contribute to spelt adaptation. Accordingly, breeding should also focus on height of spelt line stature.

### Adaptation of wheat and spelt under Mediterranean environment

Mediterranean growing conditions are characterized by extreme terminal drought and heat that require much earlier phenology (early flowering germplasm). This might explain the high correlation between DH, Yield, TKW and especially HI, that was not confounded by plant height (and accordingly DM) of the tested genotypes (Table 2). The association between DH and yield was still visible (Table 2), suggesting yield advantage for early heading lines. This superiority was prominent despite supplementary irrigation was applied at spring providing an advantage to grain filling of intermediate-late heading lines over the early modern checks. This does not resemble on-farm conditions where irrigation is not available or too costly implying much heavy GY penalties in case late heading spelt lines are grown. This is also what our records from spelt field trials have clearly shown (Figure S3).

The bread wheat cultivars are free-threshing, have a dense spike, and are semi dwarf and therefore, have a high HI (Fig. 4a). Plant height and HI are direct outcomes of the ‘green revolution’ and, of course, cannot be used exclusively to define spelt and bread wheat, leaving the spike compactness and the Hull (%) the only true visible differences between modern bread wheat and spelt. However, these two features did not correlate with each other in the present study (Table 2), and at least five lines with a speltoid spike (low SC), that were initially defined as spelt, were in fact free-threshing, and low-Hull (%) (Table 1), making the classification of spelt grains a challenging task. Trying to address this challenge, we have previously suggested that spelt breeders will use the non-free-threshing character as the main selection criteria as this is the only available clear definition for spelt^11^. The lack of complete concordance between spike morphology and Hull (%) was also found in the past^24^ and suggests that spike shape is governed by multiple genes.

The threshing character is controlled by the rachis and glume toughness^25^. An unbreakable rachis allows mechanical pressure on the glumes and soft glumes allow even easier threshing. The present analysis found that speltoid free-threshing lines have somewhat tougher glumes than modern bread wheat (Fig. 3). Curzon et al. (2019) showed that all free-threshing lines, including those with speltoid spikes, have a dominant *Q* allele in the *Q* locus, the major determinant of the threshing trait in hexaploid wheats^24^. However, additional genes are involved in controlling the threshing character^7^ and in conferring tough glumes, which might explain the tougher glumes of speltoid lines. Free-threshing lines also differ from hulled lines in GS and SW (Fig. 4a), but this differentiation had some clear exceptions and therefore cannot be used for spelt classification purposes. The separation between wheat and spelt based on threshing character was corroborated by KASP markers profiles (Fig. 2). This provides an efficient tool for authentication of spelt in breeding programs, as well as along the marketing chain, down to the household level. Interestingly, large groups of free-threshing speltoid lines, mostly from the Near-East (+ 70% of lines) occupy an intermediate position in the genetic space between the non-threshing spelt germplasm and modern bread wheat cultivars (Fig. 2). It can be assumed that these might represent spontaneous or artificial spelt-wheat introgressions and might represent a slightly separate genepool; verifying this might require additional genomic investigation. Post-harvest hulling is not required in this germplasm, highlighting its advantage for growers. Nevertheless, this group of lines, currently classified in gene banks as spelt wheat, is posing challenges in grain quality diagnostics for the milling industry. Although this challenge was at least partially addressed by Curzon et al. (2019), development of a rapid means of identifying and classifying spelt grains and flour is still required.

### Grain minerals and protein profiles of spelt under Mediterranean environment

It was suggested that common physiological and/or genetic factors control GPC and the concentrations of some mineral nutrients. Grain mineral concentration, with the exception of Zn and Na, was found to be phenotypically highly correlated with GPC (Table 3). This finding agrees with other studies^26,27^. The highest correlations observed were for S and P. The involvement of ‘S’ in protein conformation is mainly due to its role in forming disulphide bonds (S–S), which stabilize protein structure^28^. It is also known that plants tend to maintain a relatively constant ratio of organic N to organic S, even though the ratio of total N to total S can vary widely in response to N supply and fertilization^29^. The strong correlation between P and protein content was also described in an earlier study^30^ that tested winter wheat and found that GPC strongly correlated with both phytic acid and total P. Approximately 75% of the total P in the wheat grain is stored as phytic acid (myo-inositol 1, 2, 3, 4, 5, 6-hexakisphosphate), mostly in the germ and aleurone layers^31^. This molecule is a strong chelator of positively charged mineral cations such as Fe, Zn, Ca, K and Mg^32,33^ The high correlation between P and GPC may explain the strong correlations between GPC and the levels of other minerals. It has been suggested that grain Zn and Fe concentrations increase concurrently with phytate and P concentrations^32^. Thus, selection for increased GPC may result in increased grain phytic acid, subsequently resulting in high GMC. At the same time, an increase in mineral content through this pathway needs to be carefully considered, as phytic acid is an anti-nutritional factor, which prevents uptake of minerals in the gut by its chelating activity^32^. Importantly, despite this general trend, the correlation between Zn content and GPC was only on the verge of being significant (*p* < 0.068). Zn did not correlate significantly with P and other minerals, except for Fe and S (Table 3), suggesting that grain Zn content is not tightly associated with GPC.

Correlations between GY and GMC were reported in some trials^33–36^, but not others^37^. The current data generated from field assays in a Mediterranean environment showed a clear negative relationship between yield, GMC and GPC (Table 3). Calculating mineral content highlighted free-threshing line superiority and suggest that the higher GMC in low-yielding hulled spelt lines is a result of a dilution effect rather than a higher GMC per se. Therefore, comparing GMC and GPC of modern wheat varieties bred for high yield under high agronomic inputs to those of low input wheats, such as spelt, might be to some extent wrong. To minimize the effect of both environment and management on quality traits, comparisons should be drawn between wheat gene pools grown under similar conditions^38^. Moreover, comparisons must adjust and standardize for grain yield levels in order to account for the large yield differences between wheat genotypic groups (e.g., spelt *vs*. modern bread wheat). Alternatively, contrasting lines can be crossed and the outcome segregating populations subsequently scanned^39^. With that said, the overlapping distributions of bread wheat and spelt for GPC in a diverse germplasm set (Figure S2) and the high association between protein and mineral concentration discussed above suggest that spelt and bread wheat do not differ in these respects, at least not by a clear categorical definition.

### Breeding implications

The grain yield of spelt germplasm under Mediterranean environment is low compared to average grain yields of elite bread wheat varieties and to spelt yields in Europe. The highest yield achieved in the presented multiyear experiment (3 ton/ha), fell short of the spelt yields previously reported in Germany (yield range of 3–6 ton/ha)^2^. In comparison, average wheat yields 6 ton/ha and 7–8 ton/ha in northern Israel and Germany, respectively^40^. The main factor which limits spelt yields in Mediterranean environments is the late phenology and inevitable onset of terminal drought and heat at the end of the season. This yield reduction can be minimized at least partially by implementing supplemental irrigation throughout the season, but this is of course not relevant as exceed water for irrigation is in most cases not available or too costly. Therefore, early-flowering spelt lines may be a relevant breeding path, especially if the high market value of spelt grains is maintained.

KASP profiling (Fig. 2) showed that truly hulled and free-threshing spelts represent distinct gene pools, and consequently, hulled cultivars may offer some allelic diversity to bread wheat breeding programs. In addition, a notably large group of free-threshing lines which carry a speltoid spike deserves an attention. Genetically, this group occupies an intermediate position when plotted across both genetic axes (e.g., Coord. 1 and 2 in Fig. 2). Although these lines were initially defined by the gene bank curators as spelts, based on their spike morphology^12^, a Q-gene-based definition might diagnose them as non-authentic spelt varieties. This material might represent spelt-wheat historical introgression and might represent slightly separate gene pool with relevance to breeding. For example, three lines (Tomarense, 1755 and 109) with high Fe and protein in a high-yield genetic background, out of which two cultivars showed high Zn as well (Fig. 5) were identified. Although, all three were initially described as spelt and carry a speltoid spike, two lines were found to be free-threshing. Nevertheless, they can serve as a source for breeding high yielding cultivars with high protein, Zn and Fe, as suggested by others^5^.

## Conclusion

In conclusion, spelt breeding in a Mediterranean environment requires extreme phenological adjustment via introduction of *PPD-D1* photoperiod-insensitive allele into spelt genetic background. This is the major adaptive bottleneck, however importance of spring type alleles in the VRN loci should also be considered. Following this path spelt could better compete with bread wheat under short season with limited and fluctuating rainfall. However, there will be a need to follow up this intensive phenology shift because the shortening of the growing season and the dry, warm Mediterranean climate might also affect spelt grain quality via classical G x E.

## Materials and methods

### Plant material

A global diverse collection of 182 spelt lines representing the crop eco-geographic distribution was previously constructed (Curzon et al. 2019, represented in Table S1) was used for wide screening of phenology in the field. A core panel of 20 (18 spelt lines including Israeli elite modern cultivars Ruta and sheaf were chosen as checks, Table 1) was chosen for in depth phenotypic evaluation, and profiling of mineral and protein content.

### Phenotypic screening of a wide spelt collection

The wide panels of spelt genotypes were evaluated in a common garden [47 genotypes in 2015–16 (sowing date 19/11/2016) and 135 genotypes in 2016–17 experiment (sowing date 24/11/2016) seasons]. Lines were evaluated in a common garden experimental design (1 m row), for Days to heading (DH) and plant height (PH) measured as described by Chandrasekhar et al.^41^ and threshing character (THC) described in Curzon et al. (2019). Screening was conducted in a net house in Bet Dagan, Israel (31°59′38″ North, 34°49′09″ East; 40 m above sea level) in Rhodoxeralf soil. The field was treated with fungicides and pesticides to prevent development of fungal pathogens or insect pests and was weeded manually every month. Soil was fertilized 2.5 units before sowing and 5 units at seven leaf stage.

### Assessing sowing time effect on spelt phenology

A subset of the core panel (13 spelt lines and the two modern checks, marked as ‘#’ in Table S1) was sown on three separate dates (early—8/11/2015, intermediate—23/11/2015, late -12/12/2015) in order to test the impact of sowing time on spelt phenology. The field was monitored for: **days to heading** (DH) – heading date was determined at the approximate date of heading of 80% of the spikes in the plot. Relationships between the three sowing and the three heading dates for all 15 lines were calculated based on sowing date intervals (**SDI):** the later sowing date minus the earlier one (SD2-1) and (SD3-2), and heading date interval (**HDI)**: the interval between two heading dates originating two different sowing dates (HD2-1) and (HD3-2) for each line, were calculated (The later minus the earlier one).

### In depth phenotypic evaluation in the field

The core panel (n = 20, marked as * mark in Table S1) was grown (plots size of 0.5 X 0.4 m, each contained three rows) in a randomized complete block design (RCBD) experimental design (n = 4). Field was treated as described previously and did at 2016–17 season Annual rainfall was 405 mm and a 100 mm supplementary irrigation was provided at the end of March (28.3.2017) to minimize the effect of terminal drought during grain filling. The traits used for screening of the wide collection were also applied here. In addition the following traits as being recorded: Stem width (SW), Spike compactness (SC), Grain shape (GS), Dry matter (DM), Thousand-kernel weight (TKW), Hull (%), grain yield (GY), Grain protein concentration (GPC) and grain mineral concentration (GMC). Traits were measured as follows: **SW**—width of the 3^rd^ internode of three plants randomly selected from each plot, was measured using an absolute digimatic caliper (CD-6"AX, Mitutoyo, Kawasaki, Japan). Spike compactness (**SC)**—was calculated by counting the number of spikelets in each spike of four random spike samples from each plot and then dividing it by the corresponding spike length (excluding awns). **GS**—70 seeds randomly selected from each plot, were scanned with a table scanner covered with a black background box above (A4 size); the scanned pictures were analyzed using Grain scan^42^, and the majellipse that represents the approximate grain length was divided by the minellipse that represents approximate grain width, giving a measurable value for the grain shape. **DM**—the above-ground biomass (AGB) and TKW measured as described in Chandrasekhar et al.^41^. The sum of the free and hulled seeds gave the grain yield **(GY)**. The weight percentage of the peeled hulled seeds gave the Hull (%). Mean Hull (%) value was calculated for each line within block based on three row data.

### Evaluation of mineral and protein concentration

Grain protein concentration (GPC) was measured as described in Curzon et al. (2019). Grain protein concentration was measured from 20-g free-grain (not hulled) samples. Spelt samples included all core panel samples from the RCBD experiment (n = 80) and additional lines that were selected randomly from the 135 lines grown in 2016–17. In parallel, an additional 124 bread wheat samples representing twelve cultivars grown across four different environments in Israel during 2017 were also tested.

Grain mineral concentration was measured by sample of 20 g taken from each line and then ground using a laboratory mill (3100, Perten Instruments, Hägersten, Sweden); the whole wheat or spelt flour samples were analysed for minerals (barium (Ba), boron (B), calcium (Ca), copper (Cu), iron (Fe), magnesium (Mg), molybdenum (Mo), phosphorus (P), potassium (K), silicon (Si), sodium (Na), strontium (Sr), sulphur (S), zinc (Zn) concentrations (ppm) by inductively-coupled plasma mass spectrometry. Mineral content of plot (0.25m^2^) was calculated multiplying yield and minerals concentration (per gram of wheat grains).

### On field yield evaluation

In order to further assess spelt yield performances under representative Mediterranean environments, five selected lines from the core panel 1755 (PI 378480), 1152 (PI 367203), Rojo (PI 191100), White spring (PI 168,682), TAS06 were grown for three consecutive seasons (2016–17 to 2018–19), in the following types of microplots (1.6 X 7 m): (1) a common garden experiment 2016–17 in central Israel (Rehovot, Israel 34º 47′ 51′' North, 31º 54′ 20′' East; 54 m above sea level), (2) a complete randomized block design (n = 4) in 2017–18 and in 2018–19 in north of Israel (Gadash farm, 33° 10′ 48′' North, 35° 34′ 48′' East; 40 m above sea level), which had annual rainfall of 505 mm and 850 mm at 2017–2018 and 2018–2019 respectively. Upon maturity microplots were harvested with a Winter steiger plot combine (Innkreis, Austria) and kernels were weighed. Superiority index (P) was calculated for kernel yield, according to Lin and Binns^43^.

### Genetic characterization

Out of the wide collection 81 spelt lines were screened for DNA markers associated with flowering control genes related to vernalization and photoperiod (Table S2). Genotyping for *VRN-1* was done according to Yan et al. (2004) (*VRN-A1*), Milec et al.^44^ (*VRN-B1*) and Fu et al. (2005) and Muterko et al.^45^ (*VRN-D1*). Genotyping for *PPD-1* was done according to Wilhelm et al.^46^ (*PPD-A1*, insensitive allele scan and *PPD-B1*) Nishida et al. (2013) and Beales et al. (2007) *(PPD-D1*).

The core panel and additional six genotypes (PI 367,200, PI 520,066, PI 367,201, PI 367,202, PI 674,998 and TA1089) were genotyped with 83 KASP markers, representing 14 chromosomes of the A and B genomes (http://www.cerealsdb.uk.net/cerealgenomics/CerealsDB/wheat_durum_ref.php), following the description of Frankin et al. (2019). GenAlEx 6.503^47^ was used to calculate binary genetic distances between genotypes (Fig. 2). Principal coordinate analysis (PCoA) was used to illustrate genetic distances.

### Statistical analysis

Descriptive statistics of Anova and multivariate analysis (PCA) of phenotypic traits of Stem width (SW), Spike compactness (SC), Grain shape (GS), Thousand-kernel weight (TKW), Hull (%), grain yield (GY), Harvest index (HI) and mineral concentrations, Grain protein concentration (GPC) and Genotypic data was analysed using PcOA analysis following (Frankin et al. 2019). Statistical analysis of phenotypic data was preformed using the JMP13 statistical package (Pro 13, SAS Institute, Cary, NC).

## Supplementary Information

Supplementary Figures

Supplementary Tables
