# Supplementary material for: Assessing adaptive requirements and breeding potential of spelt under Mediterranean environment
Source: Sci Rep. 2021 Mar 30;11:7208. doi: 10.1038/s41598-021-86276-1 (PMC8010017; doi:10.1038/s41598-021-86276-1)
Supplement: Supplementary file 1 — Supplementary Figures [file 41598_2021_86276_MOESM1_ESM.docx]

**Supplementary files: Figures**

**Title: Assessing adaptive requirements and breeding potential of Spelt under Mediterranean environment**

Arie Y. Curzon^1, 2,^ †, Kottakota Chandrasekhar^1^, †, Kamal Nashef^1^, Shahal Abbo^2^, David J. Bonfil^3^, Ram Reifen^4^, Shimrit Bar-El^4^, On Rabinovich^5^, Asaf Avneri^2^ and Roi Ben-David^1^*

^1^Department of Vegetable and Field Crops, Institute of Plant Sciences, Agricultural Research Organization (ARO)-Volcani Center, Rishon LeZion 7528809, Israel; [arie.curzon@mail.huji.ac.il](mailto:arie.curzon@mail.huji.ac.il) (A.Y.C.); [chandrabiotech@gmail.com](mailto:chandrabiotech@gmail.com) (K.C.); [kamal@volcani.agri.gov.il](mailto:kamal@volcani.agri.gov.il) (K.N.); [roib@volcani.agri.gov.il](mailto:roib@volcani.agri.gov.il) (R.B.-D.)

^2^The Levi Eshkol School of Agriculture, The Hebrew University of Jerusalem, Rehovot 7610001, Israel; [shahal.abbo@mail.huji.ac.il](mailto:shahal.abbo@mail.huji.ac.il) (S.A.); [avneri49@gmail.com](mailto:avneri49@gmail.com) (A.A.); [arie.curzon@mail.huji.ac.il](mailto:arie.curzon@mail.huji.ac.il) (A.Y.C.).

^3^Department of Vegetable and Field Crops, Institute of Plant Sciences, Agricultural Research Organization (ARO)-Gilat Research Center, 8531100, Israel; [bonfil@volcani.agri.gov.il](mailto:bonfil@volcani.agri.gov.il) (D.J.B.)

^4^The School of Nutritional Sciences, The Robert H. Smith Faculty of Agriculture, Food and Environment, The Hebrew University of Jerusalem, Rehovot 7610001, Israel; Shimrit.Bar-El@mail.huji.ac.il (S.B.E.); [ram.reifen@mail.huji.ac.il](mailto:ram.reifen@mail.huji.ac.il) (R.R.)

^5^Northern R&D, P.O. Box 831, Kiryat Shmona 11016, Israel; [onnrab@gmail.com](mailto:onnrab@gmail.com) (O.R.)

† Contributed equally to this work

* Correspondence: [roib@volcani.agri.gov.il](mailto:roib@volcani.agri.gov.il) (R.B.-D.); Tel: +972-3-9683681; Fax: +972-3-9669642

**
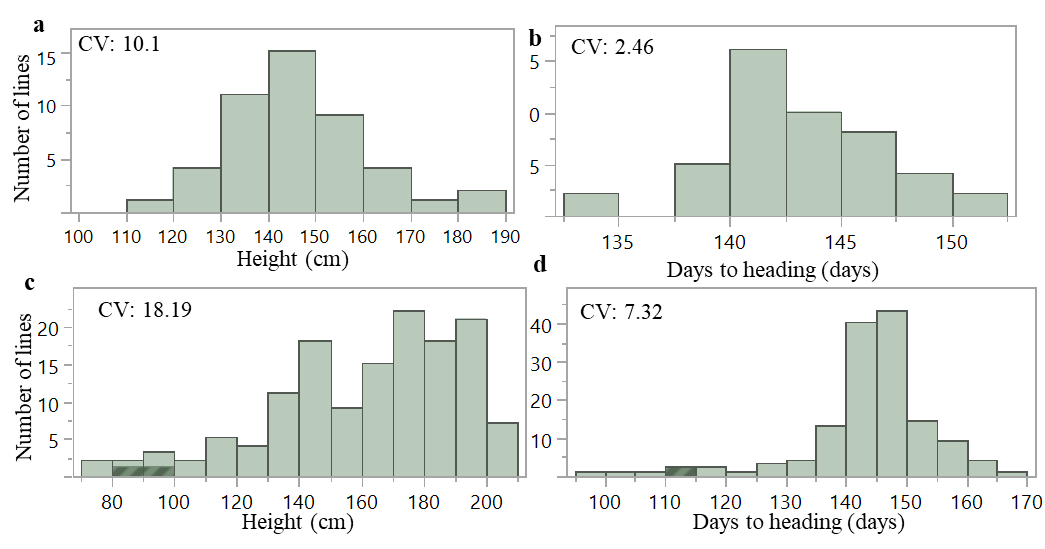
**

**Supplementary Figure S1.** Distribution of plant height and days to heading of spelt lines. (a, b) Distribution of plant height and days to heading of 47 spelt lines (CGN gene bank, see supplementary table S1) grown in common garden plots in 2015-16. (c, d**).** Distribution of plant height and days to heading for 135 spelt lines (Other than CGN lines, see supplementary table S1) and two Israeli bread wheat cultivar checks grown in 2016-17, bread wheat checks are darkened.

**
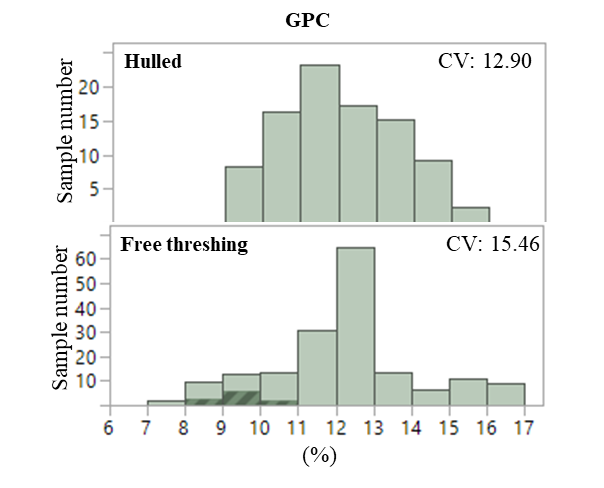
**

**Supplementary Figure 2.** Distribution of GPC among hulled (n=90) and free-threshing (n=166) grain samples. GPC measured with NIR System (6500, Foss, Hilleroed, Denmark), measures reflectance in the 400–2498 nm wavelength range at 2 nm intervals. Samples from the two Israeli bread wheat cultivar checks (n=8) grown in 2016-17 are darkened.

**Supplementary Figure S3.** Mean grain yield of spelt genotypes and superiority index. over two growing seasons. Mean (±SE) grain yield of five spelt genotypes growing for two seasons in the field a complete randomized design (n=4) in 2017-18 and 2018-19 in Gadash farm (north Israel). Superiority index was calculated based on integrated data of earlier 2016-17 season in Rehovot (central Israel, mean values not presented) and those two season. Spelt lines are 1755 (PI 378480), 1152 (PI 367203), Rojo (PI 191100), White spring (PI 168682), TAS06.
