# Supplementary material for: Assessing adaptive requirements and breeding potential of spelt under Mediterranean environment
Source: Sci Rep. 2021 Mar 30;11:7208. doi: 10.1038/s41598-021-86276-1 (PMC8010017; doi:10.1038/s41598-021-86276-1)
Supplement: Supplementary file 2 — Supplementary Tables [file 41598_2021_86276_MOESM2_ESM.docx]

**Supplementary information: Tables**

**Title:** **Assessing adaptive requirements and breeding potential of Spelt under Mediterranean environment**

Arie Y. Curzon^1, 2, †^, Kottakota Chandrasekhar ^1, †^, Kamal Nashef^1^, Shahal Abbo^2^, David J. Bonfil^3^, Ram Reifen^4^, Shimrit Bar-El^4^, On Rabinovich^5^, Asaf Avneri^2^ and Roi Ben-David^1^*****

^1^Department of Vegetable and Field Crops, Institute of Plant Sciences, Agricultural Research Organization (ARO)-Volcani Center, Rishon LeZion 7528809, Israel; [arie.curzon@mail.huji.ac.il](mailto:arie.curzon@mail.huji.ac.il) (A.Y.C.); [chandrabiotech@gmail.com](mailto:chandrabiotech@gmail.com) (K.C.); [kamal@volcani.agri.gov.il](mailto:kamal@volcani.agri.gov.il) (K.N.); [roib@volcani.agri.gov.il](mailto:roib@volcani.agri.gov.il) (R.B.-D.)

^2^The Levi Eshkol School of Agriculture, The Hebrew University of Jerusalem, Rehovot 7610001, Israel; [shahal.abbo@mail.huji.ac.il](mailto:shahal.abbo@mail.huji.ac.il) (S.A.); [avneri49@gmail.com](mailto:avneri49@gmail.com) (A.A.); [arie.curzon@mail.huji.ac.il](mailto:arie.curzon@mail.huji.ac.il) (A.Y.C.).

^3^Department of Vegetable and Field Crops, Institute of Plant Sciences, Agricultural Research Organization (ARO)-Gilat Research Center, 8531100, Israel; [bonfil@volcani.agri.gov.il](mailto:bonfil@volcani.agri.gov.il) (D.J.B.)

^4^The School of Nutritional Sciences, The Robert H. Smith Faculty of Agriculture, Food and Environment, The Hebrew University of Jerusalem, Rehovot 7610001, Israel; [Shimrit.Bar-El@mail.huji.ac.il](mailto:Shimrit.Bar-El@mail.huji.ac.il) (S.B.E.); [ram.reifen@mail.huji.ac.il](mailto:ram.reifen@mail.huji.ac.il) (R.R.)

^5^Northern R&D, P.O. Box 831, Kiryat Shmona 11016, Israel; [onnrab@gmail.com](mailto:onnrab@gmail.com) (O.R.)

^†^ Contributed equally to this work

***** Correspondence: [roib@volcani.agri.gov.il](mailto:roib@volcani.agri.gov.il) (R.B.-D.); Tel: +972-3-9683681; Fax: +972-3-9669642

**Table S1** continued

**Table S1**: Lines used in the current study, source (providers), origin and seasons. All lines belongs to the wide collection of spelt (*Triticum aestivum* ssp. spelta) except two Israeli bread wheat (*T. aestivum*) cultivars Ruta and Shefa. -: Not available. Lines marked with ***** were included in the **core panel** used for evaluation of agronomic traits and concentrations of grain minerals (GMC) and proteins (GPC). **^#^** were included the subset of core panel used to study effect of phenology on different time point sowing. NSGC USA: The National Small Grains Collection (USDA gene bank), USA, CGN: Centre for Genetic Resources, the Netherlands, TAU ISR: Lieberman Germplasm Bank, Institute for Cereal Crops Improvement, Tel-Aviv University. IPK Germany: The GenBank of the Leibniz Institute of Plant Genetics and Crop Plant Research (IPK) in Gatersleben, Germany.

| **Plant ID** | **Name** | **Source (Gene bank/institution)** | **Origin** | **Season** |
| --- | --- | --- | --- | --- |
| TAS03*^,#^ | - | Weizmann institute | unknown | 2015-16 & 2016-17 |
| TAS06*^,#^ | - | Weizmann institute | unknown | 2015-16 & 2016-17 |
| PI 608792*^,#^ | Tomarense | NSGC | Portugal | 2015-16 & 2016-17 |
| PI 355661*^,#^ | 69Z5.142 | NSGC | Italy | 2015-16 & 2016-17 |
| PI 225295*^,#^ | 109 | NSGC | Iran | 2015-16 & 2016-17 |
| PI 191617*^,#^ | 1772-14038 | NSGC | Portugal | 2015-16 & 2016-17 |
| PI 378480*^,#^ | 1755 | NSGC | Macedonia | 2015-16 & 2016-17 |
| PI 367203*^,#^ | 1152 | NSGC | Afghanistan | 2015-16 & 2016-17 |
| PI 191100* ^,#^ | Rojo | NSGC | Spain | 2015-16 & 2016-17 |
| PI 190962*^,#^ | - | NSGC | Unknown | 2015-16 & 2016-17 |
| Canadian*^,#^ | - | Unknown | Canada | 2015-16 & 2016-17 |
| Oren*^,#^ | - | Unknown | Unknown | 2015-16 & 2016-17 |
| Ruta*^,#^ | - | ARO | Israel | 2015-16 & 2016-17 |
| Shefa*^,#^ | - | Hazera seeds | Israel | 2015-16 & 2016-17 |
| PI 168682*^,#^ | White Spring (alstrum) | NSGC | Virginia, United States | 2015-16 & 2016-17 |
| CGN06533* | - | CGN | Iran | 2015-16 & 2016-17 |
| CGN08295* | - | CGN | unknown | 2015-16 & 2016-17 |
| CGN08306* | - | CGN | Azerbaijan | 2015-16 & 2016-17 |
| CGN08309* | - | CGN | Azerbaijan | 2015-16 & 2016-17 |
| sharanse* | - | TAU ISR | unknown | 2015-16 & 2016-17 |
| PI 367200 | 599 | NSGC USA | Ghowr, Afghanistan | 2016-17 |
| PI 520066 | 26867-302Y-300M-OY | NSGC USA | Italy | 2016-17 |
| PI 367201 | 600 | NSGC USA | Unknown | 2016-17 |
| PI 367202 | 625 | NSGC USA | Unknown | 2016-17 |
| PI 674998 | GE.2013-07 | TAU ISR | Unknown | 2016-17 |
| TA1089 | SCHWAB ENKORN | TAU ISR | Unknown | 2016-17 |
| CGN04208 | - | CGN | unknown | 2015-16 |
| CGN06529 | - | CGN | unknown | 2015-16 |
| CGN06530 | - | CGN | unknown | 2015-16 |
| CGN06531 | - | CGN | unknown | 2015-16 |
| CGN06532 | - | CGN | unknown | 2015-16 |
| CGN08276 | - | CGN | Germany | 2015-16 |
| CGN08277 | - | CGN | unknown | 2015-16 |
| CGN08278 | - | CGN | unknown | 2015-16 |
| CGN08279 | - | CGN | unknown | 2015-16 |
| CGN08280 | - | CGN | unknown | 2015-16 |
| CGN08281 | - | CGN | unknown | 2015-16 |
| CGN08282 | - | CGN | unknown | 2015-16 |
| CGN08283 | - | CGN | unknown | 2015-16 |
| CGN08284 | - | CGN | Sahara | 2015-16 |
| CGN08285 | - | CGN | unknown | 2015-16 |
| **Table S1** continued | | |  |  |
| **Plant ID** | **Name** | **Source (Gene bank/institution)** | **Origin** | **Season** |
| CGN08286 | - | CGN | Belgium | 2015-16 |
| CGN08288 | - | CGN | unknown | 2015-16 |
| CGN08289 | - | CGN | unknown | 2015-16 |
| CGN08290 | - | CGN | unknown | 2015-16 |
| CGN08291 | - | CGN | unknown | 2015-16 |
| CGN08292 | - | CGN | unknown | 2015-16 |
| CGN08293 | - | CGN | unknown | 2015-16 |
| CGN08294 | - | CGN | unknown | 2015-16 |
| CGN08296 | - | CGN | unknown | 2015-16 |
| CGN08297 | - | CGN | unknown | 2015-16 |
| CGN08298 | - | CGN | unknown | 2015-16 |
| CGN08299 | - | CGN | Iran | 2015-16 |
| CGN08300 | - | CGN | Sweden | 2015-16 |
| CGN08302 | - | CGN | Sahara | 2015-16 |
| CGN08303 | - | CGN | Japan | 2015-16 |
| CGN08304 | - | CGN | Iran | 2015-16 |
| CGN08305 | - | CGN | Azerbaijan | 2015-16 |
| CGN08307 | - | CGN | Azerbaijan | 2015-16 |
| CGN08308 | - | CGN | Azerbaijan | 2015-16 |
| CGN08313 | - | CGN | unknown | 2015-16 |
| CGN10409 | - | CGN | unknown | 2015-16 |
| CGN10410 | - | CGN | unknown | 2015-16 |
| CGN10412 | - | CGN | unknown | 2015-16 |
| CGN10413 | - | CGN | Iran | 2015-16 |
| CGN12269 | - | CGN | unknown | 2015-16 |
| CGN12270 | - | CGN | unknown | 2015-16 |
| CGN16075 | - | CGN | unknown | 2015-16 |
| CGN21063 | - | CGN | unknown | 2015-16 |
| PI 168681 | White Spring | NSGC USA | unknown | 2016-17 |
| PI 191392 | Arduini | NSGC USA | Ethiopia | 2016-17 |
| PI 191393 | Duhamelianum | NSGC USA | Ethiopia | 2016-17 |
| PI 191394 | Coeruleum | NSGC USA | Ethiopia | 2016-17 |
| PI 221419 | Album | NSGC USA | Serbia | 2016-17 |
| PI 272577 | I-1-553 | NSGC USA | unknown | 2016-17 |
| PI 323438 | PI 323438 | NSGC USA | Vienna, Austria | 2016-17 |
| PI 347914 | 69Z6.67 | NSGC USA | Bern, Switzerland | 2016-17 |
| PI 348448 | 69Z6.622 | NSGC USA | Oviedo, Spain | 2016-17 |
| PI 348453 | 69Z6.628 | NSGC USA | Oviedo, Spain | 2016-17 |
| PI 348455 | 69Z6.630 | NSGC USA | Oviedo, Spain | 2016-17 |
| PI 348458 | 69Z6.633 | NSGC USA | Oviedo, Spain | 2016-17 |
| PI 348460 | 69Z6.635 | NSGC USA | Oviedo, Spain | 2016-17 |
| PI 348461 | 69Z6.636 | NSGC USA | Oviedo, Spain | 2016-17 |
| PI 348464 | 69Z6.639 | NSGC USA | Oviedo, Spain | 2016-17 |
| PI 348465 | 69Z6.640 | NSGC USA | Oviedo, Spain | 2016-17 |
| PI 348478 | 69Z6.654 | NSGC USA | Oviedo, Spain | 2016-17 |
|  |  |  |  |  |
| **Table S1** continued | |  |  |  |
| **Plant ID** | **Name** | **Source (Gene bank/institution)** | **Origin** | **Season** |
| PI 348479 | 69Z6.655 | NSGC USA | Oviedo, Spain | 2016-17 |
| PI 348480 | 69Z6.656 | NSGC USA | Oviedo, Spain | 2016-17 |
| PI 348487 | 69Z6.663 | NSGC USA | Oviedo, Spain | 2016-17 |
| PI 348494 | 69Z6.670 | NSGC USA | Oviedo, Spain | 2016-17 |
| PI 348503 | 69Z6.680 | NSGC USA | Oviedo, Spain | 2016-17 |
| PI 348512 | 69Z6.689 | NSGC USA | Oviedo, Spain | 2016-17 |
| PI 348518 | 69Z6.695 | NSGC USA | Oviedo, Spain | 2016-17 |
| PI 348520 | 69Z6.697 | NSGC USA | Oviedo, Spain | 2016-17 |
| PI 348530 | 69Z6.708 | NSGC USA | Oviedo, Spain | 2016-17 |
| PI 348531 | 69Z6.709 | NSGC USA | Oviedo, Spain | 2016-17 |
| PI 348535 | 69Z6.713 | NSGC USA | Oviedo, Spain | 2016-17 |
| PI 348542 | 69Z6.720 | NSGC USA | Oviedo, Spain | 2016-17 |
| PI 348551 | 69Z6.730 | NSGC USA | Oviedo, Spain | 2016-17 |
| PI 348581 | 69Z6.761 | NSGC USA | Oviedo, Spain | 2016-17 |
| PI 348582 | 69Z6.762 | NSGC USA | Oviedo, Spain | 2016-17 |
| PI 348588 | 69Z6.768 | NSGC USA | Oviedo, Spain | 2016-17 |
| PI 348598 | 69Z6.779 | NSGC USA | Oviedo, Spain | 2016-17 |
| PI 348610 | 69Z6.791 | NSGC USA | Oviedo, Spain | 2016-17 |
| PI 348630 | 69Z6.812 | NSGC USA | Oviedo, Spain | 2016-17 |
| PI 348636 | 69Z6.818 | NSGC USA | Oviedo, Spain | 2016-17 |
| PI 348637 | 69Z6.819 | NSGC USA | Oviedo, Spain | 2016-17 |
| PI 348639 | 69Z6.821 | NSGC USA | Oviedo, Spain | 2016-17 |
| PI 348640 | 69Z6.822 | NSGC USA | Oviedo, Spain | 2016-17 |
| PI 348641 | 69Z6.823 | NSGC USA | Oviedo, Spain | 2016-17 |
| PI 348644 | 69Z6.827 | NSGC USA | Oviedo, Spain | 2016-17 |
| PI 348646 | 69Z6.829 | NSGC USA | Oviedo, Spain | 2016-17 |
| PI 348647 | 69Z6.830 | NSGC USA | Oviedo, Spain | 2016-17 |
| PI 348654 | 69Z6.837 | NSGC USA | Oviedo, Spain | 2016-17 |
| PI 348655 | 69Z6.838 | NSGC USA | Oviedo, Spain | 2016-17 |
| PI 348656 | 69Z6.839 | NSGC USA | Oviedo, Spain | 2016-17 |
| PI 348661 | 69Z6.844 | NSGC USA | Oviedo, Spain | 2016-17 |
| PI 348664 | 69Z6.847 | NSGC USA | Oviedo, Spain | 2016-17 |
| PI 348673 | 69Z6.857 | NSGC USA | Oviedo, Spain | 2016-17 |
| PI 348683 | 69Z6.867 | NSGC USA | Oviedo, Spain | 2016-17 |
| PI 348686 | 69Z6.870 | NSGC USA | Oviedo, Spain | 2016-17 |
| PI 348687 | 69Z6.871 | NSGC USA | Oviedo, Spain | 2016-17 |
| PI 348707 | 69Z6.891 | NSGC USA | Oviedo, Spain | 2016-17 |
| PI 348713 | 69Z6.897 | NSGC USA | Oviedo, Spain | 2016-17 |
| PI 348739 | 69Z6.924 | NSGC USA | Oviedo, Spain | 2016-17 |
| PI 348740 | 69Z6.925 | NSGC USA | Oviedo, Spain | 2016-17 |
| PI 348742 | 69Z6.928 | NSGC USA | Oviedo, Spain | 2016-17 |
| PI 348751 | 69Z6.937 | NSGC USA | Oviedo, Spain | 2016-17 |
| PI 348754 | 69Z6.940 | NSGC USA | Oviedo, Spain | 2016-17 |
| PI 348765 | 69Z6.952 | NSGC USA | Oviedo, Spain | 2016-17 |
| PI 348769 | 69Z6.956 | NSGC USA | Oviedo, Spain | 2016-17 |
|  |  |  |  |  |
| **Table S1** continued | |  |  |  |
| **Plant ID** | **Name** | **Source (Gene bank/institution)** | **Origin** | **Season** |
| PI 348771 | 69Z6.958 | NSGC USA | Oviedo, Spain | 2016-17 |
| PI 355564 | SK 2D | NSGC USA | Switzerland | 2016-17 |
| PI 355585 | SK 4B | NSGC USA | Switzerland | 2016-17 |
| PI 355611 | Braunschweig 1302 | NSGC USA | Lower Saxony, Germany | 2016-17 |
| PI 355618 | Braunschweig 1300 | NSGC USA | Lower Saxony, Germany | 2016-17 |
| PI 355625 | Spelta 34 | NSGC USA | Namur, Belgium | 2016-17 |
| PI 355642 | 69Z5.122 | NSGC USA | Unknown | 2016-17 |
| PI 355664 | Roter Kolben Dinkel | NSGC USA | Bavaria, Germany | 2016-17 |
| PI 355692 | BP 5 - N | NSGC USA | Rhine-Westphalia, Germany | 2016-17 |
| PI 355701 | BP 6 - N | NSGC USA | Rhine-Westphalia, Germany | 2016-17 |
| PI 367199 | 128 | NSGC USA | Harat, Afghanistan | 2016-17 |
| PI 348720 | 69Z6.905 | NSGC USA | Unknown | 2016-17 |
| PI 348580 | 69Z6.760 | NSGC USA | Unknown | 2016-17 |
| PI 225271 | 85 | NSGC USA | Unknown | 2016-17 |
| PI 469049 | MG 27212 | NSGC USA | Unknown | 2016-17 |
| PI 191392 | Arduini | NSGC USA | Unknown | 2016-17 |
| PI 190963 | Spelta Hohenheim | NSGC USA | Unknown | 2016-17 |
| PI 615299 | N15 | NSGC USA | Unknown | 2016-17 |
| TRI 3238 | - | IPK Germany | Afghanistan | 2016-17 |
| TRI 12943 | - | IPK Germany | Spain | 2016-17 |
| TRI 16898 | - | IPK Germany | Italy | 2016-17 |
| TRI 28863 | Spelz, Dinkel | IPK Germany | Iran | 2016-17 |
| TRI 5648 | - | IPK Germany | Iran | 2016-17 |
| TRI 9681 | - | IPK Germany | Morocco | 2016-17 |
| PI 295059 | Deutchland | NSGC USA | Unknown | 2016-17 |
| PI 295060 | Hinducusch | NSGC USA | Unknown | 2016-17 |
| PI 295061 | Ungarn | NSGC USA | Unknown | 2016-17 |
| PI 295062 | Italien | NSGC USA | Unknown | 2016-17 |
| PI 295063 | Ungarn | NSGC USA | Unknown | 2016-17 |
| PI 295064 | Weisser Granenspelz | NSGC USA | Unknown | 2016-17 |
| PI 295066 | Anatolien | NSGC USA | Unknown | 2016-17 |
| PI 295067 | Babenhauser Zuchtveesen | NSGC USA | Unknown | 2016-17 |
| PI 295068 | Kipperhaus Roter Spelz | NSGC USA | Unknown | 2016-17 |
| PI 295069 | Rottweiler Fruhkorn | NSGC USA | Unknown | 2016-17 |
| PI 346853 | - | NSGC USA | Unknown | 2016-17 |
| PI 378469 | 1744 | NSGC USA | Unknown | 2016-17 |
| PI 572914 | WIR 52470 | NSGC USA | Unknown | 2016-17 |
| PI 572915 | WIR 52469 | NSGC USA | Unknown | 2016-17 |
| PI 585008 | WIR 52464 | NSGC USA | Unknown | 2016-17 |
| PI 591904 | Liestal 11 | NSGC USA | Unknown | 2016-17 |
| TA1087 | CERALIO | TAU ISR | Unknown | 2016-17 |
| TA1088 | SCHWAB SPELT | TAU ISR | Unknown | 2016-17 |
| TA1090 | Oberkulmer rotc.11 | TAU ISR | Unknown | 2016-17 |
| TA1092 | 106/92/02-26 0220 | TAU ISR | Unknown | 2016-17 |
| TA1093 | LSA-13/96/02 | TAU ISR | Unknown | 2016-17 |
| TA1094 | LSA-30/96/02 | TAU ISR | Unknown | 2016-17 |
| TA-344 (2013) | Album | TAU ISR | Unknown | 2016-17 |
| 55030 | - | Israel grower | Unknown | 2016-17 |

**Table S2**. Lines genotyped for *VRN1* and *PPD1* alleles and days to heading (regardless to growing season). **-**: Not available. *PPD-D1b*: photoperiod sensitive, *PPD-D1a*: photoperiod insensitive. Vernalization alleles *vrnA1*, *VrnA1b*, *VrnB1*, *VrnB1a/b*, *VrnB1c*, *VrnD1a*, *vrnD1*.

| **Plant ID** | **Name** | **DH** | ***PPDD1*** | ***VRND1*** | ***VRNB1*** | ***VRNA1*** |
| --- | --- | --- | --- | --- | --- | --- |
| CGN08289 | - | 141 | *PPD-Db* | *vrnD1* | Vrn-B1c | *VrnA1b* |
| CGN08304 | - | 141 | *PPD-Db* | *vrnD1* | Vrn-B1c | *VrnA1b* |
| CGN10412 | - | 141 | *PPD-Db* | *vrnD1* | Vrn-B1c | *VrnA1b* |
| CGN08292 | - | 143 | *PPD-Db* | *vrnD1* | Vrn-B1c | *VrnA1b* |
| CGN08294 | - | 143 | *PPD-Db* | *vrnD1* | Vrn-B1c | *VrnA1b* |
| CGN08297 | - | 143 | *PPD-Db* | *vrnD1* | Vrn-B1c | *VrnA1b* |
| CGN12270 | - | 144 | *PPD-Db* | *vrnD1* | *VrnB1a/b* | *VrnA1b* |
| CGN08298 | - | 145 | *PPD-Db* | *vrnD1* | vrnb1 | *VrnA1b* |
| CGN08296 | - | 147 | *PPD-Db* | *vrnD1* | Vrn-B1c | *VrnA1b* |
| CGN08313 | - | 148 | *PPD-Db* | *vrnD1* | vrnb1 | *vrnA1* |
| CGN21063 | - | 148 | *PPD-Db* | *vrnD1* | Vrnb1 | *vnA1* |
| CGN08293 | - | 150 | *PPD-Db* | *vrnD1* | vrnb1 | *VrnA1b* |
| CGN08295 | - | 151 | *PPD-Db* | *vrnD1* | *VrnB1c* | *VrnA1b* |
| PI 520066 | 26867-302Y-300M-OY | 99 | *PPD-Da* | *VrnD1a* | *vrnb1* | *VrnA1b* |
| PI 367200 | 599 | 118 | *PPD-Db* | *VrnD1a* | *VrnB1a/b* | *vrnA1* |
| PI 367199 | 128 | 122 | *PPD-Db* | *VrnD1a* | *vrnb1* | *vrnA1* |
| PI 367201 | 600 | 125 | *PPD-Db* | *VrnD1a+vrnD1* | *VrnB1a/b* | *vrnA1* |
| PI 367202 | 625 | 128 | *PPD-Db* | *VrnD1a* | *VrnB1a/b* | *vrnA1* |
| PI 355701 | BP 6-N | 133 | *PPD-Db* | *vrnD1* | *VrnB1c* | *VrnA1b* |
| TRI 5648 | - | 135 | *PPD-Db* | *VrnD1a* | *vrnb1* | *VrnA1a* |
| PI 355564 | SK 2D | 137 | *PPD-Db* | *vrnD1* | *VrnB1c* | *VrnA1b* |
| PI 347914 | 69Z6.67 | 138 | *PPD-Db* | *vrnD1* | *VrnB1c* | *VrnA1b* |
| PI 348610 | 69Z6.791 | 138 | *PPD-Db* | *vrnD1* | *vrnb1* | *VrnA1b* |
| PI 348683 | 69Z6.867 | 139 | *PPD-Db* | *vrnD1* | *VrnB1a/b* | *VrnA1b* |
| PI 355585 | SK 4B | 139 | *PPD-Db* | *vrnD1* | *VrnB1c* | *VrnA1b* |
| PI 323438 | - | 140 | *PPD-Db* | *vrnD1* | *VrnB1c* | *VrnA1b* |
| PI 348646 | 69Z6.829 | 140 | *PPD-Db* | *VrnD1s* | *vrnb1* | *VrnA1b* |
| PI 348630 | 69Z6.812 | 141 | *PPD-Db* | *vrnD1* | *vrnb1* | *VrnA1b* |
| PI 348664 | 69Z6.847 | 141 | *PPD-Db* | *vrnD1* | *vrnb1* | *VrnA1b* |
| PI 348739 | 69Z6.924 | 141 | *PPD-Db* | *vrnD1* | *vrnb1* | *VrnA1b* |
| PI 348751 | 69Z6.937 | 141 | *PPD-Db* | *vrnD1* | *vrnb1* | *VrnA1b* |
| PI 225271 | 85 | 142 | *PPD-Db* | *VrnD1a* | *vrnb1* | *vrnA1* |
| PI 348542 | 69Z6.720 | 142 | *PPD-Db* | *vrnD1* | *vrnb1* | *VrnA1b* |
| PI 348673 | 69Z6.857 | 142 | *PPD-Db* | *vrnD1* | *vrnb1* | *VrnA1b* |
| PI 348769 | 69Z6.956 | 142 | *PPD-Db* | *vrnD1* | *vrnb1* | *VrnA1b* |
| PI 355664 | Roter Kolben Dinkel | 142 | *PPD-Db* | *vrnD1* | *vrnb1* | *VrnA1b* |
| PI 191392 | Arduini | 143 | *PPD-Db* | *vrnD1* | *vrnb1* | *VrnA1b* |
| PI 348494 | 69Z6.670 | 143 | *PPD-Db* | *vrnD1* | *vrnb1* | *VrnA1b* |
| PI 348535 | 69Z6.713 | 143 | *PPD-Db* | *vrnD1* | *VrnB1a/b* | *VrnA1b* |
| PI 348464 | 69Z6.639 | 144 | *PPD-Db* | *vrnD1* | *vrnb1* | *VrnA1b* |
| PI 348465 | 69Z6.640 | 144 | *PPD-Db* | *vrnD1* | *vrnb1* | *VrnA1b* |
| PI 348479 | 69Z6.655 | 144 | *PPD-Db* | *vrnD1* | *vrnb1* | *VrnA1b* |
| PI 355625 | Spelta 34 | 144 | *PPD-Db* | *vrnD1* | *VrnB1c* | *VrnA1b* |
| PI 355692 | BP 5-N | 144 | *PPD-Db* | *vrnD1* | *VrnB1a/b* | *vrnA1* |
| PI 348551 | 69Z6.730 | 145 | *PPD-Db* | *vrnD1* | *vrnb1* | *VrnA1b* |
| PI 348478 | 69Z6.654 | 146 | *PPD-Db* | *vrnD1* | *vrnb1* | *VrnA1b* |
|  |  |  |  |  |  |  |
| **Table S2** continued | |  |  |  |  |  |
| **Plant ID** | **Name** | **DH** | ***PPDD1*** | ***VRND1*** | ***VRNB1*** | ***VRNA1*** |
| PI 348582 | 69Z6.762 | 146 | *PPD-Db* | *vrnD1* | *vrnb1* | *vrnA1* |
| PI 348637 | 69Z6.819 | 146 | *PPD-Db* | *vrnD1* | *vrnb1* | *VrnA1b* |
| PI 348639 | 69Z6.821 | 146 | *PPD-Db* | *vrnD1* | *vrnb1* | *VrnA1b* |
| PI 348520 | 69Z6.697 | 147 | *PPD-Db* | *vrnD1* | *vrnb1* | *VrnA1b* |
| PI 348687 | 69Z6.871 | 147 | *PPD-Db* | *vrnD1* | *vrnb1* | *VrnA1b* |
| PI 348707 | 69Z6.891 | 147 | *PPD-Db* | *vrnD1* | *VrnB1a/b* | *VrnA1b* |
| PI 355618 | Braunschweig 1300 | 147 | *PPD-Db* | *vrnD1* | *VrnB1c* | *vrnA1* |
| PI 348588 | 69Z6.768 | 152 | *PPD-Db* | *vrnD1* | *vrnb1* | *VrnA1b* |
| PI 221419 | Album | 153 | *PPD-Db* | *vrnD1* | *VrnB1c* | *VrnA1b* |
| PI 348530 | 69Z6.708 | 153 | *PPD-Db* | *vrnD1* | *vrnb1* | *VrnA1b* |
| PI 191393 | Duhamelianum | 157 | *PPD-Db* | *vrnD1* | *vrnb1* | *VrnA1b* |
| PI 191394 | Coeruleum | 157 | *PPD-Db* | *vrnD1* | *vrnb1* | *vrnA1* |
| PI 355642 | 69Z5.122 | 164 | *PPD-Db* | *vrnD1* | *vrnb1* | *vrnA1* |
| - | TAS03 | 103 | *PPD-Da* | *VrnD1a* | *vrnb1* | *VrnA1b* |
| CGN08306 | - | 138 | *PPD-Db* | *vrnD1* | *vrnb1* | *VrnA1b* |
| CGN06533 | - | 145 | *PPD-Db* | *VrnD1a* | *vrnb1* | *VrnA1a* |
| PI 355611 | Braunschweig 1302 | 146 | *PPD-Db* | *vrnD1* | *VrnB1c* | *vrnA1* |
| TAS06 | N | 147 | *PPD-Db* | *vrnD1* | *VrnB1c* | *VrnA1b* |
| PI 355661 | 69Z5.142 | 147 | *PPD-Db* | *vrnD1* | *VrnB1c* | *VrnA1b* |
| PI 225295 | 109 | 147 | *PPD-Da* | *vrnD1* | *vrnb1* | *vrnA1* |
| PI 348458 | 69Z6.633 | 147 | *PPD-Db* | *vrnD1* | *vrnb1* | *VrnA1b* |
| PI 191617 | 1772-14038 | 148 | *PPD-Db* | *vrnD1* | *vrnb1* | *VrnA1b* |
| CGN08309 | - | 148 | *PPD-Db* | *vrnD1* | *VrnB1a/b* | *VrnA1b* |
| CGN08295 | - | 148 | *PPD-Db* | *vrnD1* | Vrn-B1c | *VrnA1b* |
| PI 378480 | 1755 | 149 | *PPD-Db* | *VrnD1s* | *vrnb1* | *VrnA1b* |
| PI 190962 | - | 150 | *PPD-Db* | *vrnD1* | *VrnB1c* | *VrnA1b* |
| - | Canadian | 150 | *PPD-Db* | *vrnD1* | *vrnb1* | *vrnA1+VrnA1b* |
| PI 191100 | Rojo | 151 | *PPD-Db* | *vrnD1* | *vrnb1* | *VrnA1b* |
| - | Shefa | 110 | *PPD-Da* | *VrnD1a* | *VrnB1a/b* | *vrnA1* |
| - | Ruta | 113 | *PPD-Da* | *VrnD1a* | *VrnB1a/b* | *vrnA1* |
| PI 367203 | 1152 | 119 | *PPD-Db* | *VrnD1a* | *VrnB1a/b* | *vrnA1* |
| PI 608792 | Tomarense | 141 | *PPD-Db* | *vrnD1* | *vrnb1+VrnB1a/b* | *VrnA1b* |
| - | Oren spelt | 165 | *PPD-Db* | *vrnD1* | *vrnb1* | *vrnA1* |
| PI 168682 | White Spring (Alstrum) | 139 | *PPD-Db* | *vrnD1* | *VrnB1c* | *VrnA1b* |
| - | Saharense | 108 | *PPD-Db* | *VrnD1a* | *vrnb1* | *VrnA1b* |

| **Table S3**: Relationship between the sowing and heading dates for the 15 lines (13 spelt and 2 bread wheat lines) grown in 2015-16 season (Bet-Dagan, Israel). (DH) for the early (1), intermediate (2) and late (3) sowing dates, the interval between sowing dates (SDI) and the interval between heading dates (HDI) for sowing periods early - intermediate (1&2) and intermediate - late (2&3). | | | | | | | |
| --- | --- | --- | --- | --- | --- | --- | --- |
| **Line** | **DH1** | **DH2** | **DH3** | **SDI (1&2)** | **HDI (1&2)** | **SDI (2&3)** | **HDI (2&3)** |
| TAS03 | 78 | 89 | 74 | 14 | 25 | 15 | 10 |
| TAS06 | 158 | 142 | 127 | 14 | -2 | 15 | 5 |
| Tomarense | 151 | 135 | 120 | 14 | -2 | 15 | 5 |
| 69Z5.142 | 156 | 142 | 127 | 14 | 0 | 15 | 4 |
| 109 | 163 | 146 | 131 | 14 | -3 | 15 | 0 |
| 1772-14038 | 163 | 147 | 132 | 14 | -2 | 15 | -1 |
| 1755 | 162 | 146 | 131 | 14 | -2 | 15 | 5 |
| 1152 | 112 | 104 | 89 | 14 | 6 | 15 | 6 |
| Rojo | 163 | 146 | 131 | 14 | -3 | 15 | 4 |
| White spring | 157 | 144 | 129 | 14 | 1 | 15 | 4 |
| 2670 | 156 | 146 | 131 | 14 | 4 | 15 | 3 |
| Canadian | 161 | 149 | 134 | 14 | 2 | 15 | -1 |
| Oren | 173 | 153 | 138 | 14 | -6 | 15 | 5 |
| Ruta | 92 | 99 | 84 | 14 | 21 | 15 | 12 |
| Shefa | 95 | 99 | 84 | 14 | 18 | 15 | 12 |

Days to heading

| **Table S4:** Comparison of mineral concentration of spelt lines versus a wheat check lines (Ruta and shefa) using the Dunnett’s method. Grain mineral  elements Boron (B), Copper (Cu), Iron (Fe), Potassium (K), Magnesium (Mg), Molybdenum (Mo), Phosphorus (P), Sulphur (S),  Silicon (Si), Strontium (Sr), Zinc (Zn). Calcium (Ca), Barium (Ba).Confidence interval is marked *<0.05 **<0.01 ***<0.001. |  |
| --- | --- |

| **Genotypes** | **B** | **Cu** | **Fe** | **K** | **Mg** | **Mo** | **P** | **S** | **Si** | **Sr** | **Zn** | **Ba** | **Ca** |
| --- | --- | --- | --- | --- | --- | --- | --- | --- | --- | --- | --- | --- | --- |
| CGN06533 | 0.88 | 3.66 | 34.89 | 4346.39** | 1216.78 | 1.14 | 3801.15** | 1098.68 | 35.85 | 2.75 | 62.93 | 3.03*** | 505.59 |
| CGN08306 | 0.86 | 3.81 | 33.6 | 3800.41 | 1209.85 | 1.05 | 3735.7* | 1121.54 | 33.18 | 1.93** | 60.86 | 3.34*** | 361.83** |
| TAS03 | 0.69* | 3.76 | 26.31 | 3599.75 | 1025.52 | 1.17 | 3276.69 | 1188.42 | 24.9 | 3.27 | 59.04 | 5.86 | 364.82** |
| CGN08295 | 1.2* | 5.75*** | 30.73 | 4173.46 | 1264.44* | 1.34 | 4094.53*** | 1175.84 | 48.97 | 1.53*** | 51.51 | 1.94*** | 295.66 |
| TAS06 | 1.18* | 5.06** | 42.85 | 4225.54* | 1409.48*** | 1.51* | 4396.07*** | 1275.52 | 42.78 | 1.65*** | 70.1 | 2.23*** | 295.67*** |
| PI 608792 | 1 | 5.76*** | 41.96 | 3942.01 | 1308.63** | 1.32 | 4073.49*** | 1270.1 | 42.24 | 2.27 | 79.23** | 3.35*** | 409.74 |
| PI 355661 | 1.23** | 5.82*** | 28.53 | 4164.65 | 1351.27*** | 0.9 | 4327.75*** | 1195.61 | 34.08 | 1.66*** | 54.72 | 1.61*** | 378.18*** |
| PI 225295 | 0.93 | 4.96** | 42.99 | 3713.58 | 1225.47 | 1.15 | 3935.01*** | 1326.8* | 36.72 | 2.44 | 79.67** | 2.63*** | 302.62*** |
| PI 191617 | 1.35*** | 5.85*** | 31.46 | 4439.44** | 1409.92*** | 0.75 | 4409.36*** | 1284.28* | 39.64 | 1.69*** | 57.33 | 2.21*** | 330.23*** |
| PI 378480 | 1.18* | 5.42*** | 48.65 | 4249.95* | 1413.06*** | 1.85*** | 4336.62*** | 1313.56* | 68.86** | 2.83 | 63.88 | 3.04*** | 402.56 |
| PI 367203 | 0.75 | 3.71 | 35.39 | 3827.95 | 1235.39* | 0.94 | 3593.78 | 963.77 | 36.71 | 2** | 50.82 | 3.84** | 464.05 |
| PI 191100 | 1 | 4.86* | 36.99 | 3600.93 | 1300.7** | 1.74*** | 3846.69** | 1234.54 | 47.75 | 2.67 | 68.39 | 4.55 | 397.52* |
| PI 168682 | 1.18* | 6.31*** | 38.89 | 4237.88* | 1358.56*** | 1.33 | 4265.3*** | 1244.21 | 63.11* | 1.37*** | 53.85 | 2.20*** | 334.48*** |
| PI 190962 | 1.25** | 5.13** | 27.1 | 4427.92** | 1374.75*** | 1.12 | 4413.97*** | 1139.28 | 38.46 | 2.26 | 52.44 | 1.96*** | 451.78** |
| Canadian | 1.26** | 4.88* | 43.35 | 4203.56* | 1391.21*** | 1.27 | 4506.44*** | 1311.87* | 51.38 | 1.68*** | 65.96 | 2.10*** | 370.15** |
| Oren spelt | 1.71*** | 6.77*** | 53.73 | 5298.35*** | 1532.49*** | 1.58** | 5094.64*** | 1455.51*** | 51.55 | 1.91** | 78.07** | 2.04*** | 442.99 |
| CGN08309 | 1.31*** | 6.62*** | 35.72 | 3945.81 | 1339.95*** | 1.5* | 4152.63*** | 1211.15 | 45.5 | 1.54*** | 55.51 | 1.12*** | 350.25*** |
| Saharense | 0.96 | 3.59 | 29.04 | 3458.99 | 1078.79 | 1.4* | 3177.29 | 970.7 | 42.61 | 1.57*** | 62.68 | 2.04*** | 293.77*** |
| Ruta | 0.93 | 3.53 | 27.41 | 3627.34 | 1057.38 | 0.75 | 3124.59 | 1027.59 | 23.03 | 2.84 | 55.01 | 5.94 | 496.91 |
| Shefa | 0.76 | 3.16 | 23.94 | 3308.25 | 842.9** | 0.97 | 2586.24* | 894.9 | 20.03 | 2.33 | 50.37 | 5.69 | 408.35 |
| Std Error | 0.06 | 0.27 | 6.15 | 130.21 | 40.66 | 0.15 | 123.68 | 57.92 | 7.9 | 0.15 | 4 | 0.39 | 22.38 |

| **Table S5**. T-test of mineral concentrations and content among and between free-threshing (F) and hulled (H) lines. Number of samples are free threshing (F): 9, Hulled (H): 11. Grain mineral elements Barium (Ba), Boron (B), Copper (Cu), Iron (Fe), Magnesium (Mg), Phosphorus (P), Potassium (K), Silicon (Si), Sodium (Na), Sulphur (S), Zinc (Zn), Grain protein concentration (GPC) and Yield (g). | | | | | | | | | |
| --- | --- | --- | --- | --- | --- | --- | --- | --- | --- |
|  |  | **Mineral concentration mg/kg** | | | | **Mineral content/Plot (0.25m^2^)** | | |  |
| Mineral | Sample group | Mean | R^2^ | t Ratio | p value | Mean | R^2^ | t Ratio | p value |
| B | F | 0.86 | 0.91 | 15.94 | <.0001 | 0.08 | 0.69 | -2.58 | 0.01 |
|  | H | 1.26 |  |  |  | 0.07 |  |  |  |
| Ba | F | 3.97 | 0.87 | -9.84 | <.0001 | 0.36 | 0.89 | -10.43 | <.0001 |
|  | H | 2.27 |  |  |  | 0.14 |  |  |  |
| Cu | F | 3.99 | 0.90 | 14.20 | <.0001 | 0.37 | 0.68 | -2.31 | 0.03 |
|  | H | 5.68 |  |  |  | 0.31 |  |  |  |
| K | F | 3736.10 | 0.85 | 9.12 | <.0001 | 341.99 | 0.77 | -6.01 | <.0001 |
|  | H | 4269.80 |  |  |  | 232.85 |  |  |  |
| Mg | F | 1133.40 | 0.89 | 13.40 | <.0001 | 104.69 | 0.78 | -4.99 | <.0001 |
|  | H | 1376.90 |  |  |  | 75.76 |  |  |  |
| Na | F | 31.40 | 0.62 | -1.49 | 0.14 | 2.85 | 0.79 | -8.35 | <.0001 |
|  | H | 28.90 |  |  |  | 1.56 |  |  |  |
| P | F | 3478.21 | 0.92 | 15.79 | <.0001 | 318.62 | 0.76 | -4.52 | <.0001 |
|  | H | 4349.45 |  |  |  | 238.26 |  |  |  |
| S | F | 1095.83 | 0.74 | 6.27 | <.0001 | 99.81 | 0.75 | -5.04 | <.0001 |
|  | H | 1258.31 |  |  |  | 69.60 |  |  |  |
| Si | F | 32.81 | 0.57 | 4.38 | <.0001 | 3.18 | 0.61 | -0.93 | 0.36 |
|  | H | 48.37 |  |  |  | 2.82 |  |  |  |
| Fe | F | 32.84 | 0.46 | 1.87 | 0.07 | 2.99 | 0.59 | -2.73 | 0.01 |
|  | H | 38.00 |  |  |  | 2.15 |  |  |  |
| Zn | F | 62.29 | 0.73 | -0.68 | 0.50 | 5.70 | 0.75 | -6.64 | <.0001 |
|  | H | 61.07 |  |  |  | 3.36 |  |  |  |
| GPC | F | 10.16 | 0.81 | 9.07 | <.0001 | 5.70 | 0.74 | -4.38 | <.0001 |
|  | H | 12.50 |  |  |  | 3.36 |  |  |  |
| Yield | F | 91.88 | 0.81 | -8.41 | <.0001 | 93.15 | 0.81 | -8.31 | <.0001 |
|  | H | 55.27 |  |  |  | 55.27 |  |  |  |
